# Genetic and Environmental Effects on BMI Fluctuation Across the Adult Life Course and Its Associations With Baseline BMI and BMI Change: An Individual‐Based Study of 14 Longitudinal Twin Cohorts

**DOI:** 10.1111/dom.71040

**Published:** 2026-06-25

**Authors:** Alvaro Obeso Fernandez, Anders Eriksson, Gabin Drouard, Aline Jelenkovic, Sari Aaltonen, Miina Ollikainen, Timothy Spector, Massimo Mangino, Kaare Christensen, Axel Skytthe, Kirsten O. Kyvik, Emanuela Medda, Corrado Fagnani, Robin P. Corley, Brooke M. Huibregtse, Virgilia Toccaceli, Finn E. Rasmussen, Per Tynelius, Sarah E. Medland, Scott D. Gordon, Glen E. Duncan, Dedra Buchwald, Patrik K. E. Magnusson, Ida K. Karlsson, Anna K. Dahl Aslan, Meike Bartels, Lannie Ligthart, Eco J. C. de Geus, Margaret Gatz, David A. Butler, Hyojin Pyun, Jeeeun Kim, Soo Ji Lee, Joohon Sung, Susanne Bruins, René Pool, Nicholas G. Martin, Dorret I. Boomsma, Jaakko Kaprio, Karri Silventoinen

**Affiliations:** ^1^ Helsinki Institute for Demography and Population Health University of Helsinki Helsinki Finland; ^2^ Department of Genetics, Physical Anthropology and Animal Physiology, Faculty of Science and Technology University of the Basque Country (UPV/EHU) Bilbao Spain; ^3^ cGEM, Institute of Genomics University of Tartu Tartu Estonia; ^4^ Institute for Molecular Medicine Finland, HiLIFE University of Helsinki Helsinki Finland; ^5^ Clinicum, Department of Psychiatry University of Helsinki Helsinki Finland; ^6^ Minerva Foundation Institute for Medical Research Helsinki Finland; ^7^ Department of Twin Research and Genetic Epidemiology King's College London UK; ^8^ The Danish Twin Registry, Institute of Public Health, Epidemiology, Biostatistics & Biodemography University of Southern Denmark Odense Denmark; ^9^ Research Unit of Clinical Genetics, Department of Clinical Research University of Southern Denmark Odense Denmark; ^10^ Odense University Hospital Odense Denmark; ^11^ Centre for Behavioural Sciences and Mental Health Istituto Superiore di Sanità Rome Italy; ^12^ Institute for Behavioral Genetics University of Colorado Boulder Colorado USA; ^13^ Department of Psychology and Neuroscience University of Colorado Boulder Colorado USA; ^14^ Department of Global Public Health Karolinska Institutet Stockholm Sweden; ^15^ QIMR Berghofer Medical Research Institute Brisbane Australia; ^16^ Washington State Twin Registry, Washington State University—Health Sciences Spokane Spokane Washington USA; ^17^ Washington State Twin Registry University of Washington Seattle Washington USA; ^18^ Department of Medical Epidemiology and Biostatistics Karolinska Institutet Stockholm Sweden; ^19^ School of Health Sciences University of Skövde Skövde Sweden; ^20^ Department of Biological Psychology Vrije Universiteit Amsterdam Amsterdam the Netherlands; ^21^ Amsterdam Public Health Research Institute Amsterdam the Netherlands; ^22^ Center for Economic and Social Research University of Southern California Los Angeles California USA; ^23^ Health and Medicine Division, the National Academies of Sciences, Engineering, and Medicine Washington DC USA; ^24^ Department of Epidemiology Seoul National University School of Public Health Seoul Korea; ^25^ Institute of Health & Environmental Seoul National University Seoul Korea; ^26^ Complex Trait Genetics, Center for Neurogenomics and Cognitive Research Vrije Universiteit Amsterdam the Netherlands

**Keywords:** body composition, cohort study, meta‐analysis, observational study

## Abstract

**Introduction:**

Body mass index (BMI) fluctuation has adverse health consequences, yet its determinants remain poorly understood. We examined genetic and environmental influences on BMI fluctuation and its associations with BMI trajectories.

**Data and Methods:**

Data from 14 longitudinal twin cohorts, including 58 311 individuals (54% women) and 22 714 complete twin pairs (9761 monozygotic), were pooled. BMI trajectories from ages 18 to 99 years were estimated using linear mixed‐effects models. BMI fluctuation was defined as the average squared residual between observed and expected BMI around individual trajectories. Genetic and environmental contributions to BMI fluctuation and its associations with baseline BMI and BMI change were assessed using structural equation modelling.

**Results:**

Additive genetic effects explained a moderate proportion of variance in BMI fluctuation (a^2^ = 0.20 in men, 0.29 in women), with the remainder attributable to unique environmental effects (e^2^ = 0.80 and 0.71). No evidence of sex‐specific genetic effects was found. Genetic contributions varied across life stages, peaking in men at ages 31–50 (a^2^ = 0.36) and in women at 51–65 (a^2^ = 0.36). Higher baseline BMI was associated with greater fluctuation, whereas greater directional BMI change was associated with less fluctuation. Higher BMI at ages 18–30 was associated with greater subsequent BMI fluctuation across later adulthood stages. Previous associations were driven by both genetic and unique environmental factors.

**Conclusions:**

BMI fluctuation was predominantly explained by unique environmental effects, with moderate heritability that varied across life stages with no evidence of sex‐specific genetic effects. Genetic and environmental factors contribute to how BMI fluctuation is associated with BMI change.

## 1 Introduction

Obesity is one of the most pressing global public health challenges of the 21st century. Between 1 and 2.5 billion adults worldwide are classified as having overweight or obesity, and the prevalence is projected to rise further [1, 2]. Obesity contributes substantially to non‐communicable diseases (NCDs), including type 2 diabetes, cardiovascular diseases, and several cancers [3], and imposes enormous health, economic, and social burdens now and in the future [4, 5]. Beyond the long‐term risks of high BMI, growing evidence indicates that fluctuations in BMI over time may independently pose health risks. Repeated cycles of weight gain and loss (‘weight cycling’) have been linked to adverse cardiometabolic outcomes, including type 2 diabetes, hypertension, cardiovascular disease, and higher overall mortality [6]. The physiological mechanisms are not fully understood, but weight instability may reflect or induce insulin resistance, elevated triglycerides, increased abdominal fat accumulation, or other metabolic disturbances, which in turn can increase cardiometabolic disease risk [7, 8, 9, 10].

Genetic influences on BMI have been shown in large‐scale twin [11] and genome‐wide association studies (GWAS) [12, 13]. Twin and family studies also indicate that genetics contribute to weight changes in adulthood, with heritability estimates of 0.05–0.29 [14]. Prior research has reported phenotypic associations between BMI and subsequent weight change, as well as the genetic and environmental contributions to these associations [14]. However, studies on the heritability of BMI fluctuation, its links with BMI and BMI change, and genetic and environmental factors underlying these associations remain limited [15]. Although BMI fluctuations may largely be driven by environmental (i.e., neighbourhood walkability and the food environment) and lifestyle (i.e., changes in diet, physical activity) factors, or life circumstances, individuals may differ genetically in their sensitivity to such perturbations. If so, heritability of BMI fluctuation would reflect inherited differences in environmental responsiveness rather than direct genetic determination of weight fluctuation, consistent with the concept of ‘variability genes’ proposed by Berg [16].

To address this gap, we applied genetic twin modelling to pooled data from 14 longitudinal twin cohorts. BMI fluctuation was defined as variability around an individual's fitted BMI trajectory, and BMI change as linear increase or decrease over time. Our aims were to (i) assess cohort‐specific genetic and environmental effects and cross‐cohort heterogeneity for BMI fluctuation; (ii) examine how these effects vary across the adult life span; and (iii) identify genetic and environmental contributions to associations of BMI fluctuation with baseline BMI and BMI change across life stages.

## 2 Data and Methods

### 2.1 Cohorts

We analysed data from the CODATwins database in collaboration with the BETTER4U consortium [17], including 14 longitudinal twin cohorts from nine countries (six European countries, the USA, South Korea, and Australia). Two cohorts included only men. Across the cohorts, height and weight were measured (16%) or self‐reported (84%), and BMI was calculated as weight in kilograms divided by height in meters squared (kg/m^2^). Twin individuals with at least three measures after age 18 were included (*N* = 61 628). Distributions of baseline BMI, BMI change, and BMI fluctuation were inspected, and participants exceeding three standard deviations (SD) from the mean in any of the variables were considered outliers and excluded (*N* = 604 for baseline BMI, *N* = 854 for BMI change, *N* = 1859 for BMI fluctuation). Outliers mostly occurred in single twins within a pair, with few pairs having both members as outliers (baseline BMI: 67 pairs; BMI change: 68 pairs; BMI fluctuation: 86 pairs), indicating extreme values were generally individual‐specific. Across cohorts, the proportion of participants excluded for any outlier ranged from 1% in the Korean Twin‐Family Register to 10% in the Queensland Twin Register, likely reflecting differences in data collection and measurement protocols. The final sample included 58 311 twin individuals (54% women) and 203 374 longitudinal BMI measurements from 22 714 complete twin pairs, of which 9761 were monozygotic (MZ), 9748 same‐sex dizygotic (SSDZ), and 2210 opposite‐sex dizygotic (OSDZ). Descriptive information for all included twin cohorts after the application of exclusion criteria is presented in Table S1. Detailed information on the number and temporal distribution of BMI measurements across cohorts, including follow‐up duration and intervals between measurements, is presented in Table S2.

We first examined BMI fluctuation within each cohort separately and then in the pooled dataset, including all participants. Next, we conducted life‐stage–specific analyses by restricting the pooled data to individuals whose first and last BMI measurements fell within the same life‐stage: young adulthood (18–30 years), early midlife (31–50 years), midlife (51–65 years) or older adulthood (≥ 66 years), defined according to previous studies [18, 19] and prior CODATwins research [14]. These analyses included 16 083 individuals and 5001 complete pairs (2483 MZ, 2069 SSDZ, 449 OSDZ). Finally, to assess how BMI in young adulthood related to subsequent BMI fluctuation, we selected participants with baseline BMI recorded at ages 18–30 and classified them into early midlife (31–50 years), midlife (51–65 years) or older adulthood (≥ 66 years) based on their final BMI measurement (*N* = 28 821 individuals; 8336 complete pairs: 3726 MZ, 3657 SSDZ, 953 OSDZ). Comparison between analytic samples is presented in Table S3, and the full selection and stratification process is shown in Figure S1.

### 2.2 Calculation of BMI Trajectories and BMI Fluctuation

BMI trajectories were estimated using linear mixed‐effects (LME) models as described elsewhere [14]. Inclusion of individual‐specific random effects captured each participant's baseline BMI (kg/m^2^) and linear rate of BMI change over time (kg/m^2^ per decade).

BMI fluctuation (kg/m^2^ per decade) was computed based on deviations from the modelled trajectory. For each individual *i*, predicted BMI at age *t* was calculated as *y*_*it*_ = *x*_*it*_ *n*_*i*_ + *m*_*i*_ + *ε*_*it*_, where *y*_*it*_ is the predicted BMI for individual *i* at age *t*, *n*_*i*_ is the individual‐specific estimated BMI change (kg/m^2^ per decade), *m*_*i*_ is the individual‐specific baseline BMI (kg/m^2^), *x*_*it*_ is the difference between age *t* and the baseline age and *ε*_*it*_ is the residual error. Squared residuals between observed and predicted values were computed across all ages, and BMI fluctuation was defined as the mean of these squared residuals. This measure captures intra‐individual dispersion around the expected trajectory and is sensitive to larger deviations. Unlike absolute residuals, which down‐weight larger deviations, and the coefficient of variation, which may confound fluctuation with BMI level, it provides a direction‐independent measure of instability. By capturing deviations in both directions from the individual‐specific trajectory, BMI fluctuation is distinct from BMI change, which reflects unidirectional linear trends. For example, an individual with a steep upward BMI change but little scatter around that trend would have low fluctuation, whereas one with a flat trajectory but large oscillations would have high fluctuation (Figure S2).

To evaluate the validity of the BMI fluctuation measure, we applied an alternative method that does not assume linearity of BMI trajectories, based on the variance of delta slope estimates between consecutive BMI measurements [20]. A sensitivity analysis at cohort‐specific and pooled levels (Table S4) showed strong agreement between the two methods, with correlations ranging from 0.73–0.91 in men and 0.70–0.95 in women across cohorts, and 0.86 in men and 0.75 in women in the pooled data.

Analyses were conducted using R (version 4.2.3) with packages lme4 (1.1–34), lmerTest (3.1–3), dplyr (1.1.4), and modelsummary (1.4.3).

### 2.3 Association Analyses

The associations between BMI fluctuation and BMI trajectories (baseline BMI and BMI change) were studied using LME models. We first conducted cohort‐specific analyses to assess these associations. BMI fluctuation was considered as the dependent variable, while baseline BMI and BMI change were considered as independent variables, with one model per predictor, and baseline age and zygosity as covariates. When examining the association between BMI change and BMI fluctuation, the model was additionally adjusted for baseline BMI. After obtaining cohort‐specific estimates, we assessed between‐cohort variability using a meta‐analysis with Cochran's *Q* test, which evaluates differences in effect estimates across cohorts beyond sampling variation alone [21].

We then conducted pooled analyses to increase statistical power and obtain overall estimates, including a cohort identifier as covariate to adjust for differences in age distribution, measurement year or region of different cohorts. The analyses proceeded in three steps. First, across the entire lifespan. Second, stratified by life stage to examine age‐specific patterns. Third, BMI at young adulthood and BMI fluctuation from this stage to subsequent life‐stages to assess whether BMI in young adulthood predicted BMI fluctuation in subsequent decades. All linear mixed‐effects models included random effects for family ID to account for the non‐independence of twin observations. Analyses were stratified by sex based on previous evidence of sex differences in BMI [11], BMI change [14], and BMI fluctuation [15].

Several sensitivity analyses were conducted using pooled data. First, smoking status and education level were included as additional covariates to determine if the observed phenotypic associations were robust to adjustment for lifestyle and socioeconomic factors that may independently influence BMI trajectories and weight changes [21, 22]. The classifications of education and smoking variables in the CODATwins database have been detailed elsewhere [23, 24]. Second, to address potential bias due to differences in the number of BMI measurements per individual, we repeated the analyses by restricting the sample to individuals with more than five BMI measurements, ensuring a denser and more reliable estimation of BMI trajectories and fluctuations. Third, to assess potential bias due to self‐reported BMI, random‐effects meta‐regression analyses were conducted using the proportion of self‐reported BMI in each cohort (see Table S5) as a moderator to examine whether the reporting source influenced the observed associations.

All statistical analyses were performed using R software (version 4.2.3) with the lme4 package (version 1.1–34). The Cochran's *Q* tests were conducted using the metafor (version 3.8.1) and meta (version 6.3.0) R packages.

### 2.4 Structural Equation Modelling

Structural equation modelling of twin data was conducted to disentangle the relative influence of genetic and environmental effects on BMI fluctuation across three levels: (i) within each cohort, with between‐cohort heterogeneity assessed using Cochran's Q test [25], (ii) in the pooled dataset without life‐stage stratification, and (iii) in the pooled data stratified by life stage [26]. Twin modelling leverages differences in genetic similarity between MZ twins, who are nearly genetically identical at the genetic sequence level, and DZ twins, who share on average half of their segregating genome [19]. Phenotypic variance in BMI fluctuation can arise from additive genetic effects (A), dominant genetic effects (D), shared environment (C), and unique environmental influences (E). Standardised covariances were set to 1.0 for A, D and C in MZ pairs and 0.5 (A), 0.25 (D), and 1.0 (C) in DZ pairs, with E uncorrelated within pairs. Because D and C cannot be estimated simultaneously in twins reared together, either an additive genetic/shared environmental/unique environmental (ACE) or additive genetic/dominance genetic/unique environmental (ADE) model was fitted, with subsequent reduction to a parsimonious additive genetic/unique environmental (AE) model if the *χ* ^2^‐distributed −2LL test indicated that dropping the C or D parameter did not significantly worsen model fit (*p* > 0.05). Intraclass correlations (ICCs) calculated as the covariance between co‐twins divided by the total variance and their 95% confidence intervals (CIs), derived from the F distribution [27], were used to assess consistency across cohorts and determine pooling feasibility. The initial full model (ACE or ADE) was compared with the AE model. Nested submodels were tested to evaluate sex differences in genetic and environmental estimates and sex‐specific genetic effects using a *χ* ^2^‐distributed −2 log‐likelihood (−2LL) test. Sex‐specific genetic effects were further assessed by estimating the genetic correlation between male and female genetic factors in DZ opposite‐sex twin pairs.

To examine the genetic and environmental contributions to the associations between BMI fluctuation and BMI trajectories, we utilised bivariate Cholesky decomposition. This method allowed us to estimate genetic (*r*_A_), shared environmental (*r*_C_), and unique environmental (*r*_E_) correlations by dividing the relevant covariance by the square root of the product of the corresponding variances [28]. Analyses were performed in R (4.2.3), fitting univariate and bivariate twin models with OpenMx (2.21.11) using the maximum likelihood estimator. Cohort heterogeneity was assessed using the metafor (3.8.1) and meta (6.3.0) packages.

## 3 Results

Table 1 presents the mean BMI fluctuation values by life stage and sex. BMI fluctuation increased progressively across life stages in both sexes, with mean values approximately tripling from young adulthood (18 to 30 years) to older adulthood (≥ 66 years) in men (from 4.75 to 13.13 kg/m^2^) and women (from 8.31 to 17.61 kg/m^2^), consistently higher in women at all life stages.

**TABLE 1:** Mean BMI fluctuation by life stage and sex in the pooled data.

Life‐stage	Men		Women	
	BMI fluctuation (kg/m^2^ per decade)		BMI fluctuation (kg/m^2^ per decade)	
	Mean	SD	Mean	SD
Young adulthood (18–30 years)	4.75	11.20	8.31	15.11
Early midlife (31–50 years)	5.75	11.80	9.80	17.83
Midlife (51–65 years)	6.98	13.93	13.12	19.53
Older adulthood (≥ 66 years)	13.10	20.32	17.62	22.71

Abbreviations: BMI, body mass index; SD, standard deviation.

Table 2 shows the associations between BMI fluctuation and BMI trajectories in the pooled data. BMI fluctuation was positively associated with baseline BMI (men: *β* = 0.31, 95% CI 0.23–0.44; women: *β* = 0.52, 95% CI 0.45–0.60) and negatively associated with BMI change (men: *β* = −0.03, 95% CI −0.04 to −0.02; women: *β* = −0.07, 95% CI −0.08 to −0.06). Each 1‐unit increase in baseline BMI corresponded to a 1.70 kg/m^2^ higher BMI fluctuation per decade in men and 2.20 kg/m^2^ in women, while each 1‐unit increase in BMI change was linked to a 0.50 kg/m^2^ lower BMI fluctuation in men and 0.40 kg/m^2^ lower in women. Life‐stage–stratified analyses showed that baseline BMI remained positively associated with BMI fluctuation across all stages, and BMI change remained negatively associated, except in men during young adulthood, where the association was not significant (Table S6). Sensitivity analyses additionally adjusting for smoking and education (Table S7) yielded similar patterns, confirming robustness to further adjustment for these covariates. Sensitivity analyses restricted to individuals with five or more BMI measurements (Table S8) showed similar patterns to those observed in the full sample, confirming the robustness of the findings to differences in measurement frequency.

**TABLE 2:** Associations of BMI fluctuation (kg/m^2^ per decade) with baseline BMI (kg/m^2^) and BMI change (kg/m^2^ per decade) in the pooled dataset by sex.

Outcomes	Men			Women		
	Estimate	95% CI		Estimate	95% CI	
		LL	UL		LL	UL
	*N* = 28 087			*N* = 30 488		
Baseline BMI	0.31	0.23	0.44	0.52	0.45	0.60
BMI change	−0.03	−0.04	−0.02	−0.07	−0.08	−0.06

*Note:* Estimates and 95% confidence intervals are derived from linear mixed‐effects models adjusted for zygosity, baseline age and cohort ID in the baseline BMI‐BMI fluctuation association and for zygosity, baseline age, baseline BMI and cohort ID in the BMI change‐BMI fluctuation association. The random effect accounting for familial clustering was added in all the models.

Abbreviations: BMI, body mass index; CI, confidence interval; LL, lower limit; UL, upper limit.

Cohort‐specific results (Figures S3 and S4 and the exact values in Table S9) indicated heterogeneity in association strength and direction across cohorts (Cochran's *Q* tests, all *p* < 0.001). Meta‐regression analyses further showed no association between the proportion of self‐reported BMI across cohorts and the effect estimates for either baseline BMI or BMI change in men or women (Table S10).

The associations between BMI at young adulthood and BMI fluctuation from young adulthood to subsequent life‐stages are shown in Table 3. Higher BMI in young adulthood was significantly associated with greater BMI fluctuation in later life‐stages in both sexes. In men, the strongest effect was from young adulthood to midlife (*β* = 0.31), followed by early midlife (*β* = 0.23) and older adulthood (*β* = 0.24). For women, the associations were consistently stronger, with the largest effect from young adulthood to midlife (*β* = 0.72), and smaller yet significant effects from young adulthood to older adulthood (*β* = 0.63) and to early midlife (*β* = 0.34).

**TABLE 3:** Associations between baseline BMI (kg/m^2^) at young adulthood and BMI fluctuation (kg/m^2^ per decade) from young adulthood to subsequent stages of life by sex.

Outcomes	Men			Women		
	Estimate	95% CI		Estimate	95% CI	
		LL	UL		LL	UL
From young adulthood to early midlife	*N* = 5330			*N* = 8081		
Baseline BMI	0.23	0.14	0.33	0.34	0.25	0.42
From young adulthood to midlife	*N* = 5484			*N* = 4114		
Baseline BMI	0.31	0.26	0.42	0.72	0.63	0.84
From young adulthood to older adulthood	*N* = 5367			*N* = 445		
Baseline BMI	0.24	0.12	0.35	0.63	0.42	0.80

*Note:* Estimates and 95% confidence intervals for the associations between BMI fluctuation (kg/m^2^ per year) from young adulthood to subsequent stages and baseline BMI (kg/m^2^) at young adulthood, stratified by sex. Analyses were conducted using linear mixed‐effects models adjusted for zygosity, baseline age, and cohort ID. A random effect for family ID was included to account for familial clustering.

Abbreviations: BMI, body mass index; CI, confidence interval; LL, lower limit; UL, upper limit.

We began genetic twin modelling by identifying the best‐fitting model using ICCs in MZ and DZ pairs (Tables S11 and S12) and comparing ACE and ADE models (Tables S13 and S14). In the pooled dataset without life‐stage stratification, the ACE model was selected as baseline (rMZ < 2rDZ) (Table S11). Stratified by life stage, the ADE model fitted best for young adulthood (rMZ > 2rDZ), while ACE was preferred for other stages (Table S12). Overall, model fit analyses confirmed the ICC results in the pooled data and most life‐stage analyses, except in young adulthood where ACE provided a slightly better fit than ADE (2LL_ACE_ < 2LL_ADE_) (Table S14). Excluding shared environmental variance had minimal impact. No sex‐specific genetic effects were detected in the pooled analysis, but they emerged in young adulthood. Accordingly, the AE model without sex‐specific effects but with separate estimates for men and women was used for BMI fluctuation in pooled analyses (Table S13) and in early midlife, midlife, and older adulthood models (Table S14). For young adulthood, the full AE model including sex‐specific effects with separate estimates for men and women was selected. Cohort‐specific results for best‐fitting model selection are presented in Tables S15 and S16.

The contributions of additive genetic and unique environmental effects to BMI fluctuation in the pooled data are shown in Table 4. Without life‐stage stratification, additive genetic effects explained a moderate proportion of variance in men (*a* ^2^ = 0.20) and women (*a* ^2^ = 0.29), while unique environmental effects accounted for most of the variance (*e* ^2^ = 0.80 and 0.71, respectively). Heritability varied across life stages: in men, it was highest in middle–late adulthood (*a* ^2^ = 0.36) and lowest in midlife (*a* ^2^ = 0.15); while in women, it peaked in midlife (*a* ^2^ = 0.36) and was lowest in older adulthood (*a* ^2^ = 0.11). Across all stages and both sexes, unique environmental effects remained predominant, ranging from 0.64 to 0.85 in men and 0.64 to 0.89 in women. We did not find evidence of sex‐specific genetic effects in the pooled data, since the genetic correlation in OSDZ pairs did not differ from the 0.5 expected for SSSD pairs (0.57 95% CI: 0.38–0.75). Cohort‐specific results for heritability and unique environmental variance are presented in Figures S5 and S6 with the exact values in Table S17.

**TABLE 4:** Heritability and relative proportion of unique environmental variance for body mass index (BMI) fluctuation rate (kg/m^2^ per year) with 95% confidence intervals, presented for the pooled data (i) not stratified by life stage and (ii) data stratified by life stage, in both cases stratified by sex.

BMI fluctuation	Men						Women					
	*a* ^2^	95% CI		*e* ^2^	95% CI		*a* ^2^	95% CI		*e* ^2^	95% CI	
		LL	UL		LL	UL		LL	UL		LL	UL
Pooled data without life‐stage stratification												
All ages	0.20	0.17	0.22	0.80	0.77	0.82	0.29	0.26	0.31	0.71	0.68	0.73
Pooled data with life‐stage stratification												
Young adulthood (18–30 years)	0.35	0.28	0.42	0.65	0.57	0.71	0.30	0.19	0.39	0.70	0.60	0.80
Early midlife (31–50 years)	0.36	0.25	0.46	0.64	0.53	0.74	0.19	0.12	0.24	0.81	0.75	0.87
Midlife (51–65 years)	0.15	0.01	0.28	0.85	0.71	0.99	0.36	0.28	0.42	0.64	0.57	0.72
Older adulthood (> 66 years)	0.24	0.11	0.36	0.76	0.63	0.88	0.11	0.01	0.23	0.89	0.76	1.00

*Note:* Full AE model was selected for BMI fluctuation when not considering life stage stratification and for young adulthood when considering life stages. AE model without sex specific genetic effect was selected for the rest of the life stages (early midlife, midlife and older adulthood).

Abbreviations: *a* ^2^, heritability (proportion of total variance explained by additive genetic effects); CI, confidence interval; *e* ^2^, proportion of total variance explained by unique environmental effects; LL, lower limit; UL, upper limit.

Finally, we examined the additive genetic and unique environmental components underlying the previous significant associations (Table 5). In the pooled data without life‐stage stratification, both genetic and environmental factors contributed to the associations between baseline BMI and BMI fluctuation in men (*r*_A_ = 0.27; *r*_E_ = 0.16) and women (*r*_A_ = 0.36; *r*_E_ = 0.21), as well as to associations with BMI change (men: *r*_A_ = −0.08, *r*_E_ = −0.06; women: *r*_A_ = 0.06, *r*_E_ = −0.05). Life‐stage–stratified analyses showed that the contributions of these components varied across stages (Table S18). Finally, the associations between young adulthood BMI and BMI fluctuation from young adulthood to subsequent stages were also influenced by both genetic and environmental factors in men (*r*_A_ = 0.27–0.58; *r*_E_ = 0.08–0.16) and women (*r*_A_ = 0.35–0.61; *r*_E_ = 0.20–0.25).

**TABLE 5:** Additive genetic and unique environmental correlations of the associations between (i) BMI fluctuation and BMI trajectories and (ii) BMI in early young adulthood (18–30 years of age) and BMI fluctuation (kg/m^2^ per year) from early young adulthood to subsequent life‐stages in the pooled data by sex.

Outcomes	Men						Women					
	*r*_A_	95% CI		*r*_E_	95% CI		*r*_A_	95% CI		*r*_E_	95% CI	
		LL	UL		LL	UL		UL	LL		LL	UL
Pooled data without life‐stage stratification												
Baseline BMI	0.27	0.23	0.31	0.16	0.13	0.19	0.36	0.33	0.39	0.21	0.19	0.24
BMI change	−0.08	−0.13	−0.04	−0.06	−0.08	−0.03	0.06	0.02	0.10	−0.05	−0.07	−0.04
Baseline BMI at young adulthood–BMI fluctuation from young adulthood to subsequent stages												
From young adulthood to early midlife	0.27	0.17	0.37	0.15	0.08	0.22	0.35	0.29	0.42	0.20	0.15	0.24
From young adulthood to midlife	0.29	0.16	0.44	0.16	0.08	0.24	0.52	0.43	0.61	0.24	0.17	0.31
From young adulthood to older adulthood	0.58	0.38	1.00	0.08	0.01	0.15	0.61	0.36	0.85	0.25	0.01	0.48

*Note:* Pairwise correlations between (i) BMI fluctuation and BMI trajectories without life‐stage stratification and (ii) baseline BMI at young adulthood and BMI fluctuation from young adulthood to subsequent life‐stages were carried out. They are summarised with correlation estimates besides their confidence intervals.

Abbreviations: BMI, body mass index; CI, confidence interval; LL, lower limit; *r*_A_, additive genetic correlation; *r*_E_, specific environmental correlation; UL, upper limit.

## 4 Discussion

In this large study of pooled twin cohorts, baseline BMI was positively associated with BMI fluctuation, whereas BMI change showed a negative association in both men and women. The BMI fluctuation increased from younger to older stages in men and women, although in women the BMI fluctuation was higher in all stages. Individuals with higher initial BMI experienced greater fluctuation over time, while those with greater BMI changes exhibited less, suggesting BMI instability may be characteristic of those entering adulthood with higher body mass whose BMI does not follow a steady upward trend. Analyses of young adulthood supported this pattern, showing that higher BMI in this stage was consistently associated with greater BMI fluctuation across later life stages. The associations were strongest from young adulthood to midlife, particularly among women, indicating midlife may be a period of increased vulnerability to weight instability among individuals with overweight. These patterns likely reflect age‐related factors such as metabolic adaptations, lifestyle transitions, and health behaviours, as well as sex‐specific biological (e.g., hormonal changes, reproductive factors) and social (e.g., gendered dieting patterns) influences on weight regulation. When stratified by life stage, the positive association between baseline BMI and fluctuation persisted across all ages, peaking in middle–late adulthood, whereas the inverse association with BMI change remained generally consistent but was weakest or absent among men in young adulthood. The results align with a previous Finnish twin study showing that higher BMI in adolescence and early adulthood predicted greater subsequent fluctuation [15]. Importantly, these associations remained robust in sensitivity analyses adjusting for smoking and education, indicating the relationships are not driven by these potential confounders. Similar results were obtained when restricting analyses to individuals with five or more BMI measurements, supporting the robustness of the findings to variation in measurement frequency and indicating that sparse data did not drive the observed associations.

One possible explanation for why individuals with higher baseline BMI experience greater BMI fluctuation later in adulthood is that excess adiposity is associated with physiological changes that destabilise long‐term weight regulation. Higher BMI is linked to metabolic disturbances, including insulin resistance [7], elevated triglycerides [9], and increased abdominal fat accumulation [9, 29], which impair metabolic flexibility and may cause short‐term shifts in energy balance. Furthermore, higher BMI and related metabolic dysregulation increase vulnerability to type 2 diabetes [8, 10, 30], a condition characterised by impaired glucose homeostasis and disrupted appetite and energy regulation. Together, these mechanisms may make individuals with higher initial BMI more prone to cycles of weight gain and loss, contributing to greater BMI fluctuation across adulthood. Clinically, higher BMI in young adulthood may indicate risk for long‐term weight instability, highlighting the importance of sustainable weight management. Furthermore, individuals with higher BMI carry greater fat mass, which is more metabolically variable than lean mass, providing greater biological capacity for weight gain and loss. This physiological asymmetry may partly explain the association between baseline BMI and fluctuation, but it does not fully account for our findings, as it is attenuated in older adulthood, suggesting that mechanisms beyond fat mass become increasingly relevant with age.

Cohort‐specific analyses showed generally positive associations between baseline BMI and BMI fluctuation, although the magnitude of effects varied across cohorts and between sexes. Associations with BMI change were mostly negative, with some weak or nonsignificant estimates. Overall, this pattern indicates that while the direction of associations was broadly consistent across cohorts, substantial heterogeneity in effect size was present, as also reflected in the high *I* ^2^ values. This suggests that differences between cohorts primarily reflect variation in the strength of associations rather than fundamentally conflicting findings. Such heterogeneity is likely driven by differences in sample size, follow‐up duration, age structure, and cohort‐specific measurement conditions. Importantly, meta‐regression analyses indicated that the proportion of self‐reported BMI across cohorts did not explain between‐cohort variability in effect estimates, suggesting that measurement source is unlikely to be a major driver of the observed heterogeneity or the main findings.

Variation in BMI fluctuation was influenced by both additive genetic and unique environmental factors, with the latter accounting for most of the variance. These heritability estimates were lower than those typically reported for overall adult BMI (60%–80%) [11] but comparable to estimates for BMI change in the same database [14]. Genetic contributions varied across adulthood, peaking in middle–late adulthood for men and in midlife for women, although unique environmental effects remained dominant. Heritability estimates for BMI fluctuation were higher in the life‐stage–restricted sample compared with the full pooled sample. In men, higher monozygotic twin correlations with stable dizygotic correlations resulted in a shift from an ACE to an ADE model, whereas results in women remained largely unchanged. These differences likely reflect changes in variance structure within age‐restricted analyses rather than substantive alterations in genetic architecture (see Tables S19, S20 and S21). Cohort‐specific analyses showed heterogeneity in men for both effects and in women for unique environmental effects. These findings suggest that genetic predisposition may shape susceptibility to BMI fluctuation, potentially via metabolic or behavioural pathways, consistent with the notion of variability genes modulating individual responsiveness to environmental change [16]. Notably, while the DNA sequence itself is stable, genetic expression is not; evidence suggests that the sets of genes influencing BMI shift across the lifespan [11, 31], which may partly explain the variation in heritability estimates across life stages observed in our data. Nevertheless, environmental and lifestyle factors (e.g., pressure to lose weight and dieting) play a larger role, emphasising the importance of individual‐specific behaviours and contexts in shaping weight instability across adulthood. We did not find evidence of sex‐specific genetic factors consistent with broader evidence that qualitative sex differences in genetic architecture are rare across complex traits [32]. Nevertheless, the number of OSDZ twins was low in our data, increasing sample error when estimating this effect.

Both additive genetic and unique environmental factors underlie the associations between baseline BMI, BMI change, and BMI fluctuation. This suggests that BMI fluctuation partly reflects stable individual differences in genetic liability to higher body mass, but also individual‐specific experiences that influence weight regulation over time. The increasing importance of unique environmental factors in later adulthood may indicate that accumulated life experiences, health conditions, or behavioural factors become more central drivers of weight instability with age. In older adulthood specifically, age‐related loss of lean body mass (sarcopenia) and disease‐related weight changes may introduce additional sources of BMI variability independently of adiposity. This is consistent with the marked increase in mean BMI fluctuation observed in older adulthood, values approximately tripled compared with young adulthood in both sexes, and with the attenuation of the phenotypic association between baseline BMI and BMI fluctuation at this life stage. In contrast, the more intermittent contributions observed for BMI change suggest that dynamic weight gain processes may operate differently across life stages, potentially reflecting varying biological and lifestyle influences from emerging adulthood to later life.

A key strength of this study is the large, diverse twin sample with follow‐ups from young adulthood to older adulthood spanning more than six decades and including up to 13 repeated BMI measurements per individual. This longitudinal design allowed examination of genetic and environmental contributions to BMI fluctuation across life stages, as well as phenotypic, genetic, and environmental correlations between BMI trajectories and BMI fluctuation. Pooled data from multiple international twin cohorts provided substantial statistical power and enabled cross‐cohort comparisons. Furthermore, comparison of the main method used for estimation of BMI fluctuation and an alternative method based on the variance of consecutive slope estimates showed strong correlations between them, suggesting the main findings are not driven by the choice of definition. However, cohorts mainly represent high‐income countries in Europe, North America, Asia, and Australia, limiting generalisability to populations with predominantly Western lifestyles. Additionally, 84% of height and weight data were self‐reported, and a higher proportion of measured data would improve reliability. Detailed information on potential BMI fluctuation modifiers, such as diet, physical activity, reproductive history, and lifestyle factors beyond smoking and education, was limited. BMI, while correlating with fat mass and body fat percentage and predicting obesity risk [33, 34], does not fully capture body composition or fat distribution, nor does it distinguish between fluctuations driven by fat mass versus lean mass changes. Fat mass is more responsive to environmental changes, while lean mass is more stable but subject to age‐related decline [35, 36]. Future studies incorporating direct body composition measures such as dual‐energy X‐ray absorptiometry would allow formal disentanglement of fat and lean mass contributions to BMI fluctuation across the lifespan, particularly in older adulthood where sarcopenia may confound interpretation.

In summary, BMI fluctuation was modestly influenced by genetic effects but predominantly shaped by unique environmental factors, with heritability lower than typically reported for overall BMI. Individuals with higher baseline BMI, especially in young adulthood, experienced greater BMI fluctuation later in life, with stronger associations in women. This suggests that BMI instability mainly reflects individual‐specific environmental and behavioural influences rather than genetics. Clinically, young adulthood BMI may help identify those at risk for long‐term weight instability, highlighting the importance of promoting sustainable weight management during this critical period.

## Author Contributions

A.O.F. and K.S. designed the study. S.A., M.O., T.S., M.M., K.C., A.S., K.O.K., E.M., C.F., R.P.C., B.M.H., V.T., F.E.R., P.T., S.E.M., S.D.G., G.D., D.B., P.K.E.M., I.K.K., A.K.D.A., M.B., L.L., E.J.C.G., M.G., D.B., H.P., J.K., S.J.L., J.S., S.B., R.P., N.G.M., D.B. and J.K. collected the original data files. K.S., J.K., A.J. and A.O.F. pooled the data together. A.O.F. conducted the analyses and drafted the manuscript. A.E., G.D., A.J., S.A., M.O., T.S., M.M., K.C., A.S., K.O.K., E.M., C.F., R.P.C., B.M.H., V.T., F.E.R., P.T., S.E.M., S.D.G., G.D., D.B., P.K.E.M., I.K.K., A.K.D.A., M.B., L.L., E.J.C.G., M.G., D.B., H.P., J.K., S.J.L., J.S., S.B., R.P., N.G.M., D.B., J.K. and K.S. revised the manuscript. All authors approved the final version and agreed to be accountable for all aspects of the work in ensuring that questions related to the accuracy or integrity of any part of the work are appropriately investigated and resolved.

## Funding

This work was supported by the Chronic Disease Research Foundation, the Medical Research Council Laboratory of Molecular Biology, the Swiss State Secretariat for Education, Research and Innovation (SERI), the MagW/ZonMW, (912‐10‐020, 400‐05‐717, 451‐04‐034, 463‐06‐001, 480‐04‐004, 904‐61‐090, 904‐61‐193, 985‐10‐002, Addiction‐31160008, Middelgroot‐911‐09‐032, Spinozapremie 56‐464‐14192), the Clinical Center, R21 AG039572, the Wellcome Trust, the National Institute for Health and Care Research, the VU University's Institute for Health and Care Research, EMGO+, the European Research Council (ERC 230374), the Versus Arthritis, the NIHR Clinical Research Network North West London, the Terveyden Tutkimuksen Toimikunta (100499, 118555, 141054, 205585, 264146, 308248), the Avera Institute for Human Genetics, the Danish Agency for Science and Higher Education, the Centre for Behavioural Sciences and Mental Health, the Velux Fonden, the Horizon Europe Research and Innovation programme (101080117), the Zoe Ltd, the Research Council for Health and Disease, the Research council of Finland Centre of Excellence in Complex Disease Genetics (352792), the European Union Horizon 2020, the Centre of Research Excellence Grant (1079102), the National Institute of Health (RC2 HL103416), the Wellcome Leap Dynamic Resilience Programme, the National Institute of Health US (P01 AG08761), the NIHR Barts Biomedical Research Centre, Queen Mary University of London, the Forskningsrådet om Hälsa, Arbetsliv och Välfärd (2017‐00641), the National Institute of Alcohol Abuse and Alcoholism (AA‐00145, AA‐09203, AA‐12502, AA015416, AA15416), the UK Research and Innovation (UKRI) (10093560, 10106435) and the Global Research Network Program of the National Research Foundation (NRF 2011‐220‐E00006).

## Ethics Statement

The pooled analysis was approved by the ethical board of the Department of Public Health, University of Helsinki. The data collection procedures of participating twin cohorts were approved by local ethical boards following the regulation in each country. Only anonymised data were delivered to the data management center at University of Helsinki.

## Conflicts of Interest

The authors declare no conflicts of interest.

## Supporting information


**Figure S1:** Flow chart illustrating the selection and inclusion process of participants in the current study.
**Figure S2:** Conceptual illustration of BMI change and BMI fluctuation in two.
**Figure S3:** Association between BMI trajectories and BMI fluctuation by twin cohort in men.
**Figure S4:** Association between BMI trajectories and BMI fluctuation by twin cohort in women.
**Figure S5:** Cohort‐specific additive genetic (a^2^) and unique environmental (e^2^) effects of BMI fluctuation in men.
**Figure S6:** Cohort‐specific additive genetic (a^2^) and unique environmental (e^2^) effects of BMI fluctuation in women.
**Table S1:** Descriptive information on the included databases after application of exclusion criteria.
**Table S2:** Distribution and frequency of BMI measurements across twin cohorts included in the study.
**Table S3:** Comparison of general characteristics and BMI related measures (Baseline BMI, BMI change and BMI fluctuation) across life‐stage and pooled analytic samples.
**Table S4:** Correlations between BMI fluctuation obtained through the main model of the study and the comparison model (variance of delta slopes) by sex at cohort‐specific level and pooled data.
**Table S5:** Proportion of self‐reported BMI by cohort.
**Table S6:** Associations of BMI fluctuation (kg/m^2^ per decade) with baseline BMI (kg/m^2^) and BMI change (kg/m^2^ per decade) in the pooled dataset with life‐stage stratification, by sex.
**Table S7:** Sensitivity analysis of the associations of BMI fluctuation (kg/m^2^ per decade) with baseline BMI (kg/m^2^) and BMI change (kg/m^2^ per decade) in the pooled dataset with and without life‐stage stratification, by sex.
**Table S8:** Sensitivity analysis: associations between BMI fluctuation and BMI trajectories restricted to individuals with ≥ 5 BMI measurements.
**Table S9:** Cohort‐specific associations of BMI fluctuation (kg/m^2^ per year) with baseline BMI (kg/m^2^) and BMI change (kg/m^2^ per year), stratified by sex.
**Table S10:** Meta‐regression analysis of the effect of self‐reported BMI proportion on study estimates.
**Table S11:** Intraclass correlations for body mass index (BMI) fluctuation (kg/m^2^ per year) in pooled data without life‐stage stratification.
**Table S12:** Intraclass correlations for body mass index (BMI) fluctuation (kg/m^2^ per year) in pooled data with life‐stage stratification.
**Table S13:** Model fit statistics of body mass index (BMI) fluctuation (kg/m^2^ per year) in pooled data without life‐stage stratification.
**Table S14:** Model fit statistics of body mass index (BMI) fluctuation (kg/m^2^ per year) in a pooled data with life‐stage stratification.
**Table S15:** Intraclass correlations (and 95% confidence interval) for body mass index (BMI) fluctuation (kg/m^2^ per year) in cohort specific data by sex and zygosity.
**Table S16:** Model fit statistics of body mass index (BMI) fluctuation (kg/m^2^ per year) in cohort specific data.
**Table S17:** Heritability and relative proportion of unique environmental variance for body mass index (BMI) fluctuation rate (kg/m^2^ per year) with 95% confidence intervals, presented for each cohort and stratified by sex.
**Table S18:** Additive genetic and unique environmental correlations of the associations between BMI fluctuation and BMI trajectories with life‐stage stratification in the pooled data by sex.
**Table S19:** Comparison of intraclass correlations for body mass index (BMI) fluctuation (kg/m^2^ per year) between the full pooled sample and the life‐stage–restricted sample.
**Table S20:** Comparison of model fit statistics of body mass index (BMI) fluctuation (kg/m^2^ per year) between the full pooled sample and the life‐stage–restricted sample.
**Table S21:** Comparison of Heritability and relative proportion of unique environmental variance for body mass index (BMI) fluctuation rate (kg/m^2^ per year) between full pooled sample and the life‐stage–restricted sample in both cases stratified by sex.

## Data Availability

The data used in this study is owned by the third parties (the individual twin cohorts) and made available to this study on the condition that they will be used only in this meta‐analysis. The data can be used based on the same principles as those used in this study (more information from Karri Silventoinen karri.silventoinen@helsinki.fi).
