# Supplementary material for: Genetic and Environmental Effects on BMI Fluctuation Across the Adult Life Course and Its Associations With Baseline BMI and BMI Change: An Individual‐Based Study of 14 Longitudinal Twin Cohorts
Source: Diabetes Obes Metab. 2026 Jun 25;28(9):8418–27. doi: 10.1111/dom.71040 (PMC13448893; doi:10.1111/dom.71040)
Supplement: Supplementary file 1 [file DOM-28-8418-s001.docx]

**Supplementary Figure 1.** Flow chart illustrating the selection and inclusion process of participants in the current study.

**Caption**: The study included 148,370 individuals from cohorts with repeated BMI measurements. (1) Participants with fewer than three BMI measurements were excluded. (2) We examined the distribution of baseline BMI, BMI change, and BMI fluctuation, excluding values exceeding three standard deviations from the mean. These cleaned data were used for both pooled and cohort-specific analyses. In the first set of analyses, comparisons were conducted with and without life stage stratification. In the life stage–stratified analysis, two approaches were applied: the first included individuals with data specific to each life stage (young adulthood, middle–late adulthood, middle age and old age), and the second included individuals with a first BMI measurement in young adulthood and BMI fluctuation tracked through subsequent stages. Individual-level associations were analyzed using linear mixed-effects (LME) models. Finally, univariate and bivariate structural equation modeling was conducted using complete twin pairs. **Abbreviations**: BMI: Body mass index.

**Supplementary Figure 2.** Conceptual illustration of BMI change and BMI fluctuation in two hypothetical individuals.

**Caption:** Panel A shows an individual with high BMI change and low BMI fluctuation, characterised by a steep upward trajectory with observed values closely following the fitted trend. Panel B shows an individual with low BMI change and high BMI fluctuation, characterised by a nearly flat trajectory with observed values deviating substantially in both directions. The solid line represents the individual-specific fitted BMI trajectory estimated from a linear mixed-effects model. Dashed lines represent residuals (deviations of observed from predicted BMI values). BMI fluctuation was defined as the mean squared residual around the fitted trajectory. **Abbreviations:** BMI: body mass index

**Supplementary Figure 3.** Association between BMI trajectories and BMI fluctuation by twin cohort in men.

**Caption:** Forest plots showing the association between BMI trajectories (left baseline BMI and right BMI change) and BMI fluctuation in men across multiple twin cohorts. Each dot represents the point estimate of the association for a specific cohort, with horizontal lines indicating 95% confidence intervals. Cochran’s Q test indicates significant heterogeneity across cohorts (baseline BMI: Q = 230.56, I^2^ = 95.22 %, p = 3.22e-43; BMI change: Q = 175.03, I^2^ = 93.71 %, p = 1.08e-31). **Abbreviations:** BMI: body mass index; CI: confidence interval.

**Supplementary Figure 4.** Association between BMI trajectories and BMI fluctuation by twin cohort in women.

**Caption:** Forest plots showing the association between BMI trajectories (left baseline BMI and right BMI change) and BMI fluctuation in women across multiple twin cohorts. Each dot represents the point estimate of the association for a specific cohort, with horizontal lines indicating 95% confidence intervals. Cochran’s Q test indicates significant heterogeneity across cohorts (baseline BMI: Q = 400.56, I^2^ = 97.75 %, p = 1.03e-80; BMI change: Q = 167.94, I^2^ = 94.64 %, p = 1.65e-31). **Abbreviations:** BMI: body mass index; CI: confidence interval.

**Supplementary Figure 5.** Cohort-specific additive genetic (a^2^) and unique environmental (e^2^) effects of BMI fluctuation in men.

**Caption:** Forest plots showing the proportion of variance in traits explained by additive genetic effects (a^2^, left) and unique environmental effects (e^2^, right) across multiple twin cohorts in men. Blue circles represent point estimates, and horizontal lines indicate 95% confidence intervals. Pooled estimates from the meta-analysis are shown in the lower part of the figure. Cochran’s Q test indicates significant heterogeneity across cohorts (a^2^: Q=129.85, I^2^=96.58%, p=2.24e−21; e^2^: Q = 128.08, I^2^ = 94.62 %, p = 5.03e-21). **Abbreviations:** CI: confidence interval; a^2^: heritability (proportion of total variance explained by additive genetic effects); e^2^: proportion of total variance explained by unique environmental effects.

**Supplementary Figure 6.** Cohort-specific additive genetic (a^2^) and unique environmental (e^2^) effects of BMI fluctuation in women.

**Caption:** Forest plots showing the proportion of variance in traits explained by additive genetic effects (a^2^, left) and unique environmental effects (e^2^, right) across multiple twin cohorts in women. Blue circles represent point estimates, and horizontal lines indicate 95% confidence intervals. Pooled estimates from the meta-analysis are shown in the lower part of the figure. Cochran’s Q test indicates significant heterogeneity across cohorts only for unique environmental effects (a^2^: Q=18.88, I^2^=21.34%, p=0.06; e^2^: Q = 26.49, I^2^ = 58.65 %, p = 5.48e-03). **Abbreviations:** CI: confidence interval; a^2^: heritability (proportion of total variance explained by additive genetic effects); e^2^: proportion of total variance explained by unique environmental effects.

**Supplementary table 1.** Descriptive information on the included databases after application of exclusion criteria.

| **Cohort** | **General information** | | | **Zygosity** | | | | **Variables of interest** | | | | | | | |
| --- | --- | --- | --- | --- | --- | --- | --- | --- | --- | --- | --- | --- | --- | --- | --- |
|  | **Age range** | **N** | **% Women** | **Complete twin pairs** | **MZ** | **DZ** | | **Men** | | | | **Women** | | | |
|  |  |  |  |  |  | **SSDZ** | **OSDZ** | **Mean BMI baseline (SD)** | **Mean BMI change (SD)** | **Mean**  **BMI fluctuation (SD)** | **Mean baseline age**  **(SD)** | **Mean BMI baseline (SD)** | **Mean BMI change (SD)** | **Mean**  **BMI fluctuation (SD)** | **Mean baseline age**  **(SD)** |
| *Europe* |  |  |  |  |  |  |  |  |  |  |  |  |  |  |  |
| Danish Twin Cohort | 18-99 | 8,757 | 53% | 2,541 | 998 | 905 | 638 | 24.58 (2.72) | 0.10 (0.05) | 2.06 (2.83) | 46.59 (17.37) | 22.63 (3.07) | 0.11 (0.06) | 2.41 (3.13) | 45.27 (18.02) |
| Finnish Old Cohort | 18-99 | 12,216 | 54% | 4,940 | 1,702 | 3,237 | 0 | 23.21 (2.71) | 0.10 (0.05) | 1.88  (2.56) | 28.32 (7.40) | 21.42 (2.65) | 0.12 (0.06) | 1.96 (2.61) | 27.94 (7.51) |
| FinnTwin16 | 18-37 | 3,267 | 56% | 1,262 | 462 | 409 | 391 | 21.74 (2.35) | 0.13 (0.04) | 1.75 (2.39) | 18.64  (0.47) | 20.61 (2.25) | 0.13 (0.06) | 1.82 (2.56) | 18.58 (0.49) |
| FinnTwin12 | 18-39 | 608 | 60% | 302 | 147 | 108 | 47 | 23.83 (3.10) | 0.19 (0.22) | 2.37 (2.51) | 18.00 (0.00) | 22.40 (3.76) | 0.20 (0.25) | 2.41 (2.58) | 18.00 (0.00) |
| Italy Twin Registry | 18-78 | 25 | 67% | 109 | 59 | 25 | 25 | 25.56 (3.13) | 0.10 (0.03) | 2.19 (2.70) | 48.34 (15.89) | 23.21 (3.55) | 0.11 (0.05) | 2.11 (2.64) | 45.44 (15.72) |
| Netherlands Twin Register | 18-91 | 5,129 | 66% | 2,149 | 1,188 | 554 | 407 | 22.32 (2.78) | 0.10 (0.04) | 2.89 (3.38) | 27.63 (13.23) | 21.83  (2.84) | 0.11 (0.06) | 3.14 (3.51) | 29.70 (13.63) |
| Swedish Twin Registry | 18-99 | 6,790 | 65% | 2,156 | 790 | 1,366 | 0 | 24.49 (2.50) | 0.06 (0.04) | 2.43 (3.10) | 48.02 (8.68) | 24.02  (2.97) | 0.08 (0.06) | 2.67 (3.35) | 49.36 (8.73) |
| Swedish Young Male Twin Study | 18-30 | 1,402 | 0% | 598 | 350 | 248 | 0 | 21.50 (2.37) | 0.11 (0.02) | 1.72 (2.44) | 18.18 (0.43) | - | - | - | - |
| Twins UK | 18-89 | 3,661 | 91% | 1,625 | 841 | 778 | 6 | 24.82 (2.90) | 0.08 (0.04) | 3.31 (3.50) | 49.77 (14.11) | 23.54  (3.13) | 0.10 (0.06) | 3.71 (3.81) | 46.70 (11.68) |
| *North-America* |  |  |  |  |  |  |  |  |  |  |  |  |  |  |  |
| Colorado Twin Registry | 18-34 | 282 | 56% | 115 | 67 | 30 | 18 | 22.29 (2.67) | 0.12 (0.03) | 2.24 (2.91) | 18.28 (0.45) | 20.75 (2.51) | 0.15 (0.05) | 2.17 (2.83) | 18.34 (0.47) |
| NAS-NRC Twin Registry | 18-82 | 7,436 | 0% | 2,314 | 1,216 | 1,073 | 0 | 22.06 (2.35) | 0.08 (0.04) | 2.84 (3.25) | 20.92 (4.87) | - | - | - | - |
| Washington State Twin Registry | 18-97 | 2,526 | 67% | 1,284 | 819 | 286 | 179 | 24.83 (3.16) | 0.10 (0.04) | 2.47 (3.14) | 40.33 (18.22) | 23.50  (3.32) | 0.12 (0.06) | 2.76 (3.39) | 37.48 (16.56) |
| *Asia* |  |  |  |  |  |  |  |  |  |  |  |  |  |  |  |
| Korea Twin-Family Register | 21-79 | 397 | 72% | 182 | 152 | 30 | 0 | 24.22  (2.41) | 0.08 (0.03) | 2.06 (2.74) | 40.67 (8.56) | 22.19  (2.79) | 0.09 (0.04) | 1.91 (2.57) | 39.01 (7.43) |
| *Australia* |  |  |  |  |  |  |  |  |  |  |  |  |  |  |  |
| Queensland Twin Register | 18-92 | 5,815 | 61% | 2,167 | 970 | 699 | 498 | 23.24  (2.58) | 0.11 (0.05) | 2.89 (3.37) | 28.41 (10.01) | 21.87  (2.59) | 0.12 (0.07) | 3.08 (3.52) | 30.12 (10.93) |
| *All world* |  |  |  |  |  |  |  |  |  |  |  |  |  |  |  |
| Pooled data | 18-99 | 58,311 | 53% | 22,714 | 9,761 | 9,748 | 2,210 | 23.10 (2.72) | 0.09 (0.04) | 2.39 (3.03) | 35.56 (13.74) | 21.94 (2.84) | 0.11 (0.06) | 2.72 (3.25) | 34.75 (14.03) |

**Abbreviations:** N: Number of individuals; MZ: Monozygotic; DZ: Dizygotic; SSDZ: Same sex dizygotic; OSDZ: opposite sex dizygotic; SD: Standard deviation; UK: United Kingdom; NAS-NRC: National academy of science- National research council.

**Supplementary Table 2.** Distribution and frequency of BMI measurements across twin cohorts included in the study.

| **Cohorts** | **Min BMI count** | **Max BMI count** | **Median BMI count** | **Mean follow up years** | **Mean years between BMI measures** | **% of Individuals with** | | |
| --- | --- | --- | --- | --- | --- | --- | --- | --- |
|  |  |  |  |  |  | **3 BMI measures** | **4-5 BMI measures** | **≥ 6 BMI measures** |
| *Europe* | | | | | | | | |
| Danish Twin Cohort | 3 | 6 | 3 | 12.80 | 6.10 | 81.40 | 18.50 | 0.10 |
| Finnish Old Cohort | 3 | 4 | 3 | 27.20 | 11.20 | 60.70 | 39.30 | 0.00 |
| FinnTwin16 | 3 | 4 | 3 | 15.40 | 7.70 | 9.8 | 0.20 | 0.00 |
| FinnTwin12 | 3 | 3 | 3 | 19.80 | 8.90 | 100.00 | 0.00 | 0.00 |
| Italy Twin Registry | 3 | 4 | 3 | 5.8 | 2.80 | 3.10 | 6.90 | 0.00 |
| Netherlands Twin Register | 3 | 13 | 5 | 12.10 | 3.10 | 21.70 | 35.90 | 36.90 |
| Swedish Twin Registry | 3 | 4 | 3 | 19.60 | 9.10 | 89.00 | 11.00 | 0.00 |
| Swedish Young Male Twin Study | 3 | 3 | 3 | 7.60 | 3.80 | 100.00 | 0.00 | 0.00 |
| Twins UK | 3 | 8 | 4 | 8.60 | 2.80 | 33.30 | 49.80 | 16.80 |
| *North America* | | | | | | | | |
| Colorado Twin Registry | 3 | 3 | 3 | 10.80 | 5.40 | 100.00 | 0.00 | 0.00 |
| NAS-NRC Twin Registry | 3 | 4 | 3 | 48.80 | 21.20 | 64.90 | 35.10 | 0.00 |
| Washington State Twin Registry | 3 | 6 | 3 | 8.90 | 3.60 | 67.30 | 28.70 | 4.00 |
| *Asia* | | | | | | | | |
| Korea Twin-Family Register | 3 | 4 | 3 | 6.00 | 2.90 | 94.00 | 6.00 | 0.00 |
| *Australia* | | | | | | | | |
| Queensland Twin Register | 3 | 9 | 3 | 19.90 | 7.60 | 55.80 | 40.50 | 3.70 |
| *All World* | | | | | | | | |
| Pooled data | 3 | 13 | 3 | 21.2 | 8.9 | 66.10 | 28.80 | 5.10 |

**Caption:** The table summarizes the number and timing of BMI measurements across cohorts included in the analyses providing an overview of the temporal structure and density of longitudinal BMI measurements used to derive BMI trajectories and fluctuation. **Abbreviations:** BMI: body mass index; Min: minimum; Max: maximum; UK: United Kingdom; NAS-NRC: National Academy of Sciences–National Research Council.

**Supplementary Table 3.** Comparison of general characteristics and BMI related measures (Baseline BMI, BMI change and BMI fluctuation) across life-stage and pooled analytic samples.

| **Sample by analysis** | **N** | **% Women** | **Baseline age** | | **Baseline BMI**  **(kg/m^2^)** | | **BMI change**  **(kg/m^2^ per decade)** | | **BMI fluctuation**  **(kg/m² per decade)** | |
| --- | --- | --- | --- | --- | --- | --- | --- | --- | --- | --- |
|  |  |  | **mean** | **SD** | **mean** | **SD** | **mean** | **SD** | **mean** | **SD** |
| Pooled data | 58,311 | 53% | 33.50 | 15.35 | 22.73 | 2.96 | 0.11 | 0.06 | 6.60 | 10.34 |
| Life-stage specific | 16,083 | 60% | 42.07 | 18.93 | 23.69 | 3.55 | 0.11 | 0.06 | 9.85 | 17.45 |
| Young adulthood→ subsequent life-stages | 28,821 | 44% | 22.19 | 3.83 | 21.84 | 2.65 | 0.12 | 0.07 | 8.24 | 14.57 |

**Abbreviations:** SD: standard deviation; BMI: Body mass index; N: Number of individuals

**Supplementary table 4:** Correlations between BMI fluctuation obtained through the main model of the study and the comparison model (variance of delta slopes) by sex at cohort-specific level and pooled data.

| **Cohorts** | **Men** | | | **Women** | | |
| --- | --- | --- | --- | --- | --- | --- |
|  | **r** | **95% CI** | | **r** | **95% CI** | |
|  |  | **LL** | **UL** |  | **LL** | **UL** |
| *Europe* | | | | | | |
| Danish Twin Cohort | 0.76 | 0.74 | 0.78 | 0.79 | 0.77 | 0.80 |
| Finnish Old Cohort | 0.76 | 0.74 | 0.78 | 0.81 | 0.79 | 0.83 |
| FinnTwin16 | 0.85 | 0.82 | 0.88 | 0.80 | 0.78 | 0.82 |
| FinnTwin12 | 0.91 | 0.88 | 0.93 | 0.95 | 0.93 | 0.97 |
| Italy Twin Registry | 0.73 | 0.50 | 0.88 | 0.73 | 0.45 | 0.87 |
| Netherlands Twin Register | 0.74 | 0.57 | 0.87 | 0.84 | 0.77 | 0.90 |
| Swedish Twin Registry | 0.81 | 0.77 | 0.85 | 0.75 | 0.73 | 0.77 |
| Swedish Young Male Twin Study | 0.78 | 0.74 | 0.82 | – | – | – |
| Twins UK | 0.75 | 0.67 | 0.81 | 0.77 | 0.75 | 0.79 |
| *North America* | | | | | | |
| Colorado Twin Registry | 0.80 | 0.69 | 0.89 | 0.77 | 0.67 | 0.85 |
| NAS-NRC Twin Registry | 0.75 | 0.73 | 0.77 | – | – | – |
| Washington State Twin Registry | 0.83 | 0.78 | 0.88 | 0.70 | 0.67 | 0.73 |
| *Asia* | | | | | | |
| Korea Twin-Family Register | 0.73 | 0.57 | 0.87 | 0.74 | 0.67 | 0.80 |
| *Australia* | | | | | | |
| Queensland Twin Register | 0.74 | 0.71 | 0.77 | 0.76 | 0.74 | 0.78 |
| *All world* | | | | | | |
| Pooled data | 0.86 | 0.85 | 0.87 | 0.75 | 0.74 | 0.76 |

**Abbreviations:** BMI: Body mass index; LB: Lower bound; UB: Upper bound; UK: United Kingdom; NAS-NRC: National academy of science- National research council

**Supplementary Table 5.** Proportion of self-reported BMI by cohort

| **Cohorts** | **% of Self-reported BMI** |
| --- | --- |
| *Europe* | |
| Danish Twin Cohort | 80% |
| Finnish Old Cohort | 100% |
| FinnTwin16 | 100% |
| FinnTwin12 | 100% |
| Italy Twin Registry | 80% |
| Netherlands Twin Register | 86% |
| Swedish Twin Registry | 80% |
| Swedish Young Male Twin Study | 100% |
| Twins UK | 65% |
| *North America* | |
| Colorado Twin Registry | 99% |
| NAS-NRC Twin Registry | 65% |
| Washington State Twin Registry | 100% |
| *Asia* | |
| Korea Twin-Family Register | 0% |
| *Australia* | |
| Queensland Twin Register | 90% |
| *All world* | |
| Pooled data | 86% |

**Abbreviations:** BMI: body mass index; UK: United Kingdom; NAS-NRC: National academy of science- National research council

**Supplementary Table 6.** Associations of BMI fluctuation (kg/m^2^ per decade) with baseline BMI (kg/m^2^) and BMI change (kg/m^2^ per decade) in the pooled dataset with life-stage stratification, by sex.

| **Outcomes** | **Men** | | | **Women** | | |
| --- | --- | --- | --- | --- | --- | --- |
|  | **Estimate** | **95% CI** | | **Estimate** | **95% CI** | |
|  |  | **LL** | **UL** |  | **LL** | **UL** |
| *Young adulthood*  *(18-30 years)* | N= 2,327 | | | N= 1,539 | | |
| Baseline BMI | 0.61 | 0.52 | 0.70 | 0.53 | 0.41 | 0.64 |
| BMI change | 8.18e-03 | -1.37e-03 | 0.01 | -0.02 | -0.04 | -8.88e-03 |
| *Early midlife (31-50 years)* | N= 1,947 | | | N= 4,014 | | |
| Baseline BMI | 0.63 | 0.53 | 0.72 | 0.52 | 0.43 | 0.61 |
| BMI change | -0.04 | -0.06 | -0.03 | -0.06 | -0.07 | -0.05 |
| *Midlife*  *(51-65 years)* | N= 920 | | | N= 2,361 | | |
| Baseline BMI | 0.43 | 0.32 | 0.54 | 0.34 | 0.21 | 0.43 |
| BMI change | -0.06 | -0.07 | -0.05 | -0.05 | -0.06 | -0.04 |
| *Older adulthood*  *(≥66 years)* | N= 1,261 | | | N= 1,712 | | |
| Baseline BMI | 0.24 | 0.13 | 0.31 | 0.33 | 0.26 | 0.41 |
| BMI change | -0.04 | -0.05 | -0.03 | -0.05 | -0.06 | -0.04 |

**Caption:** Estimates and 95% confidence intervals are derived from linear mixed-effects models adjusted for zygosity, baseline age and cohort ID in the baseline BMI-BMI fluctuation association and for zygosity, baseline age, baseline BMI and cohort ID in the BMI change-BMI fluctuation association. The random effect accounting for familial clustering was added in all the models. **Abbreviations:** BMI: body mass index; CI: confidence Interval; LL: lower limit; UL: upper limit.

**Supplementary Table 7.** Sensitivity analysis of the associations of BMI fluctuation (kg/m^2^ per decade) with baseline BMI (kg/m^2^) and BMI change (kg/m^2^ per decade) in the pooled dataset with and without life-stage stratification, by sex.

| **Outcomes** | **Men** | | | **Women** | | | | |
| --- | --- | --- | --- | --- | --- | --- | --- | --- |
|  | **Estimate** | **95% CI** | | **Estimate** | | **95% CI** | | |
|  |  | **LL** | **UL** |  |  | **LL** | | **UL** |
| No life-stage stratification | | | | | | | | |
| *All ages* | N= 27,859 | | | N= 30,082 | | | | |
| Baseline BMI | 0.27 | 0.20 | 0.34 | 0.64 | 0.50 | | 0.79 | |
| BMI change | -0.02 | -0.03 | -0.01 | -0.06 | -0.10 | | -0.03 | |
| Life-stage stratification | | | | | | | | |
| *Young adulthood*  *(18-30 years)* | N= 2,285 | | | N= 1,472 | | | | |
| Baseline BMI | 0.47 | 0.04 | 0.99 | 1.17 | | 0.70 | | 1.59 |
| BMI change | -0.06 | -0.11 | 0.01 | -0.03 | | -0.05 | | -0.01 |
| *Early midlife (31-50 years)* | N= 1,900 | | | N= 3,700 | | | | |
| Baseline BMI | 1.12 | 0.46 | 1.80 | 0.73 | | 0.20 | | 1.26 |
| BMI change | -0.11 | -0.19 | -0.02 | -0.16 | | -0.20 | | -0.02 |
| *Midlife*  *(51-65 years)* | N= 885 | | | N= 2,147 | | | | |
| Baseline BMI | 0.62 | 0.01 | 1.19 | 1.26 | | 0.47 | | 2.07 |
| BMI change | -0.14 | -0.22 | -0.01 | -0.20 | | -0.27 | | -0.14 |
| *Older adulthood*  *(≥66 years)* | N= 1,170 | | | N= 1,522 | | | | |
| Baseline BMI | 1.44 | 0.87 | 2.00 | 0.91 | | 0.46 | | 1.40 |
| BMI change | -0.06 | -0.12 | -0.02 | -0.09 | | -0.17 | | -0.01 |

**Caption:** Estimates and 95% confidence intervals are derived from linear mixed-effects models adjusted for baseline age, smoking status, education level and cohort ID in the baseline BMI-BMI fluctuation association and for baseline age, baseline BMI, smoking status, education level and cohort ID in the BMI change-BMI fluctuation association. The random effect accounting for familial clustering was added in all the models. **Abbreviations:** BMI: body mass index; CI: confidence Interval; LL: lower limit; UL: upper limit.

**Supplementary Table 8.** Sensitivity analysis: associations between BMI fluctuation and BMI trajectories restricted to individuals with ≥5 BMI measurements.

| **Outcomes** | **Men** | | | **Women** | | |
| --- | --- | --- | --- | --- | --- | --- |
|  | **Estimate** | **95% CI** | | **Estimate** | **95% CI** | |
|  |  | **LL** | **UL** |  | **LL** | **UL** |
|  | N= 1,505 | | | N= 4,512 | | |
| Baseline BMI | 0.13 | 0.07 | 0.18 | 0.22 | 0.19 | 0.25 |
| BMI change | -0.02 | -0.03 | -0.01 | -0.03 | -0.05 | -0.01 |

**Caption:** This table presents results from sensitivity analyses assessing the robustness of the associations between BMI fluctuation and BMI trajectories when restricting the sample to individuals with five or more BMI measurements. Estimates are shown separately for men and women. BMI fluctuation was modelled as the outcome, with baseline BMI and BMI change included as predictors in separate models. Models were adjusted for baseline age, zygosity, and (for BMI change models) baseline BMI. Estimates are displayed along with their 95% confidence intervals. **Abbreviations:** BMI: body mass index; CI: confidence interval; LL: lower limit; UL: upper limit.

**Supplementary Table 9.** Cohort-specific associations of BMI fluctuation (kg/m² per year) with baseline BMI (kg/m²) and BMI change (kg/m² per year), stratified by sex.

| **Outcomes** | **Men** | | | **Women** | | |
| --- | --- | --- | --- | --- | --- | --- |
|  | **Estimate** | **95% CI** | | **Estimate** | **95% CI** | |
|  |  | **LL** | **UL** |  | **LL** | **UL** |
| *Europe* | | | | | | |
| *Danish Twin Cohort* | N= 4,119 | | | N= 4,636 | | |
| Baseline BMI | 0.42 | 0.32 | 0.51 | 0.52 | 0.43 | 0.64 |
| BMI change | -0.04 | -0.05 | -0.03 | -0.09 | -0.10 | -0.07 |
| *Finnish old cohort* | N= 5,655 | | | N= 6,585 | | |
| Baseline BMI | 0.84 | 0.75 | 0.93 | 1.03 | 0.94 | 1.12 |
| BMI change | -0.10 | -0.12 | -0.08 | -0.11 | -0.13 | -0.08 |
| *FinnTwin16* | N= 1,422 | | | N= 1,854 | | |
| Baseline BMI | 1.02 | 0.84 | 1.22 | 1.14 | 0.93 | 1.20 |
| BMI change | 0.04 | 2.78e-03 | 0.08 | -0.10 | -0.15 | -0.05 |
| *FinnTwin12* | N= 243 | | | N= 365 | | |
| Baseline BMI | 0.52 | 0.31 | 0.83 | 0.74 | 0.15 | 1.32 |
| BMI change | -0.04 | -0.08 | -0.02 | -0.06 | -0.09 | -0.04 |
| *Italy Twin Registry* | N= 84 | | | N= 167 | | |
| Baseline BMI | -0.14 | -1.03 | 0.71 | 0.62 | 0.08 | 1.23 |
| BMI change | -0.08 | -0.16 | -6.03e-03 | 0.05 | -0.01 | 0.12 |
| *Netherlands Twin Register* | N= 1,758 | | | N= 3,371 | | |
| Baseline BMI | 0.24 | 0.14 | 0.33 | 0.32 | 0.27 | 0.38 |
| BMI change | 0.01 | -3.31e-03 | 0.02 | -0.06 | -0.07 | -0.04 |
| *Swedish Twin Registry* | N= 2,345 | | | N= 4,445 | | |
| Baseline BMI | 0.42 | 0.24 | 0.56 | 0.54 | 0.47 | 0.63 |
| BMI change | -0.11 | -0.13 | -0.10 | -0.12 | -0.13 | -0.11 |
| *Swedish Young Male Twin Study* | N= 1,402 | | | N= 0 | | |
| Baseline BMI | 0.72 | 0.54 | 0.93 | - | - | - |
| BMI change | 0.01 | -6.41e-03 | 0.03 | - | - | - |
| *TwinsUK* | N= 331 | | | N= 3,330 | | |
| Baseline BMI | 0.25 | 0.03 | 0.51 | 0.31 | 0.24 | 0.45 |
| BMI change | -0.04 | -0.06 | -0.01 | -0.06 | -0.07 | -0.05 |
| *North America* | | | | | | |
| *Colorado Twin Registry* | N= 125 | | | N= 157 | | |
| Baseline BMI | 0.32 | -0.11 | 0.70 | 1.04 | 0.64 | 1.43 |
| BMI change | -0.01 | -0.08 | 0.06 | 0.27 | 0.16 | 0.38 |
| *NAS-NRC Twin Registry* | N= 7,436 | | | N= 0 | | |
| Baseline BMI | 0.24 | 0.19 | 0.29 | - | - | - |
| BMI change | -0.01 | -0.02 | -1.20e-03 | - | - | - |
| *Washington State Twin Registry* | N= 827 | | | N= 1,699 | | |
| Baseline BMI | 0.51 | 0.36 | 0.67 | 0.52 | 0.43 | 0.70 |
| BMI change | -0.09 | -0.16 | -0.01 | -0.09 | -0.11 | -0.07 |
| *Asia* | | | | | | |
| *Korea Twin-Family Register* | N= 113 | | | N= 284 | | |
| Baseline BMI | 0.92 | 0.43 | 1.52 | 0.73 | 0.32 | 1.04 |
| BMI change | -0.13 | -0.17 | -0.09 | -0.18 | -0.22 | -0.14 |
| *Australia* | | | | | | |
| *Queensland Twin Register* | N= 2,281 | | | N= 3,534 | | |
| Baseline BMI | 0.24 | 0.10 | 0.51 | 0.30 | 0.22 | 0.40 |
| BMI change | -1.89e-03 | -0.01 | 0.01 | -0.02 | -0.04 | -6.02e-03 |
| *Test of homogeneity* | | | | | | |
|  | Cochran's Q | I^2^ | p-value | Cochran's Q | I^2^ | p-value |
| Baseline BMI | 230.56 | 95.22 % | 3.22e-43 | 400.56 | 97.75 % | 1.03e-80 |
| BMI change | 175.03 | 93.71 % | 1.08e-31 | 167.94 | 94.64 % | 1.65e-31 |

**Caption:** Estimates and 95% confidence intervals are shown for cohort-specific associations of BMI fluctuation with baseline BMI (models adjusted for zygosity and baseline age) and with BMI change (models adjusted for zygosity, baseline age, and baseline BMI). Homogeneity test results (Cochran’s Q, I², and p-values) are reported to indicate the presence of heterogeneity across cohorts. **Abbreviations:** BMI: body mass index; CI: confidence Interval; LL: lower limit; UL: upper limit; UK: United Kingdom; NAS-NRC: National academy of science- National research council; I^2^: I square statistic.

**Supplementary Table 10.** Meta-regression analysis of the effect of self-reported BMI proportion on study estimates.

| **Outcomes** | **Men** | | | | **Women** | | | |
| --- | --- | --- | --- | --- | --- | --- | --- | --- |
|  | **β** | **95% CI** | | **p value** | **β** | **95% CI** | | **p value** |
|  |  | **LL** | **UL** |  |  | **LL** | **UL** |  |
| Baseline BMI | 0.02 | -0.02 | 0.06 | 0.33 | 0.01 | -0.07 | 0.08 | 0.86 |
| BMI change | 0.01 | -0.01 | 0.02 | 0.57 | 0.01 | -0.02 | 0.02 | 0.80 |

**Caption:** Meta-regression coefficients represent the change in effect estimate per 10% increase in the proportion of self-reported BMI across cohorts. No significant associations were observed, indicating that reporting source did not influence the results. **Abbreviations:** BMI: body mass index; CI: confidence interval; LL: lower limit; UL: upper limit.

**Supplementary Table 11.** Intraclass correlations for body mass index (BMI) fluctuation (kg/m^2^ per year) in pooled data without life-stage stratification.

| **BMI fluctuation** | **Men** | | | | | | **Women** | | | | | | **Opposite sex dizygotic (OSDZ)** | | |
| --- | --- | --- | --- | --- | --- | --- | --- | --- | --- | --- | --- | --- | --- | --- | --- |
|  | **MZ** | | | **DZ** | | | **MZ** | | | **DZ** | | |  |  |  |
|  | **r** | **95%CI** | | **r** | **95%CI** | | **r** | **95%CI** | | **r** | **95%CI** | | **r** | **95%CI** | |
|  |  | **LL** | **UL** |  | **LL** | **UL** |  | **LL** | **UL** |  | **LL** | **UL** |  | **LL** | **UL** |
| *All ages* | N= 4,156 | | | N= 4,301 | | | N= 5,519 | | | N= 5,394 | | | N= 2,164 | | |
|  | 0.18 | 0.15 | 0.21 | 0.11 | 0.08 | 0.14 | 0.26 | 0.24 | 0.29 | 0.21 | 0.19 | 0.24 | 0.13 | 0.09 | 0.17 |

**Caption**: Intraclass correlations for BMI fluctuation in pooled data without life-stage stratification are summarized by sex and zygosity with correlation coefficients besides their 95% confidence interval. **Abbreviations:** BMI, body mass index; CI, confidence interval; LL, lower limit; UL, upper limit; r: correlation coefficient; MZ: monozygotic; DZ: same sex dizygotic; OSDZ: opposite sex dizygotic; N: Number of complete twin pairs.

**Supplementary Table 12.** Intraclass correlations for body mass index (BMI) fluctuation (kg/m^2^ per year) in pooled data with life-stage stratification.

| **BMI fluctuation** | **Men** | | | | | | **Women** | | | | | | **Opposite sex dizygotic (OSDZ)** | | |
| --- | --- | --- | --- | --- | --- | --- | --- | --- | --- | --- | --- | --- | --- | --- | --- |
|  | **MZ** | | | **DZ** | | | **MZ** | | | **DZ** | | |  |  |  |
|  | **r** | **95%CI** | | **r** | **95%CI** | | **r** | **95%CI** | | **r** | **95%CI** | | **r** | **95%CI** | |
|  |  | **LL** | **UL** |  | **LL** | **UL** |  | **LL** | **UL** |  | **LL** | **UL** |  | **LL** | **UL** |
| *Young adulthood*  *(18-30 years)* | N= 486 | | | N= 320 | | | N= 306 | | | N= 136 | | | N= 151 | | |
|  | 0.38 | 0.30 | 0.45 | 0.05 | -0.05 | 0.16 | 0.30 | 0.20 | 0.40 | 0.05 | -0.11 | 0.22 | 0.05 | -0.10 | 0.20 |
| *early midlife*  *(31-50 years)* | N= 268 | | | N= 252 | | | N= 710 | | | N= 553 | | | N= 167 | | |
|  | 0.37 | 0.26 | 0.47 | 0.07 | -0.05 | 0.19 | 0.15 | 0.07 | 0.22 | 0.25 | 0.17 | 0.32 | -0.01 | -0.165 | 0.138 |
| *Midlife*  *(51-65 years)* | N= 133 | | | N= 108 | | | N= 417 | | | N= 385 | | | N= 97 | | |
|  | 0.14 | -0.02 | 0.31 | 0.07 | -0.11 | 0.25 | 0.32 | 0.23 | 0.40 | 0.27 | 0.17 | 0.36 | 0.03 | -0.16 | 0.23 |
| *Older adulthood*  *(>66 years)* | N= 140 | | | N= 122 | | | N= 193 | | | N= 193 | | | N= 34 | | |
|  | 0.28 | 0.12 | 0.43 | 0.07 | -0.10 | 0.24 | 0.09 | -0.04 | 0.23 | 0.07 | -0.06 | 0.21 | 0.01 | -0.33 | 0.34 |

**Caption**: Intraclass correlations for BMI fluctuation in pooled data with life-stage stratification across young adulthood (18–30 years), middle-late adulthood (31–50 years), middle age (51–65 years), and old age (>66 years), are summarized by sex and zygosity with correlation coefficients besides their 95% confidence interval. **Abbreviations:** BMI, body mass index; CI, confidence interval; LL, lower limit; UL, upper limit; r: correlation coefficient; MZ: monozygotic; DZ: same sex dizygotic; OSDZ: opposite sex dizygotic; N: Number of complete twin pairs.

**Supplementary Table 13.** Model fit statistics of body mass index (BMI) fluctuation (kg/m^2^ per year) in pooled data without life-stage stratification.

| **BMI fluctuation** | **Full ACE model (reference model)** | | **Full ADE model** | | **Full AE model (1)** | | **Selected model without sex-specific genetic effect (2)** | | **Selected model with same parameter estimates for men and women (3)** | |
| --- | --- | --- | --- | --- | --- | --- | --- | --- | --- | --- |
|  | **-2LL** | **d.f** | **-2LL** | **d.f** | **Δ -2 LL** | **p value** | **Δ -2 LL** | **p value** | **Δ -2 LL** | **p value** |
| *All ages* | 357546.3 | 43049 | 357580.2 | 43048 | 3.38 | 0.44 | 0.49 | 0.48 | 71.53 | 1.36e-15 |

**Caption:** Model comparisons were first conducted between the full ACE and ADE models based on the −2 log-likelihood (−2LL) values. The best-fitting model from this comparison (ACE) was then tested against the simpler AE model. Finally, the selected model was compared with (i) the same model without sex-specific genetic effects and (ii) the same model without sex differentiation. The table displays the −2 log-likelihood (−2LL) and degrees of freedom (df) for the reference model, as well as the differences in −2LL (Δ−2LL) and corresponding p-values for the compared models. **Abbreviations:** Δ (change); ACE (additive genetic/ shared environment/ unique environment) model; ADE (additive genetic/ dominance genetic/ unique environment); AE (additive genetic/ unique environment) model. (1) Compared to full ACE model (Δ d.f. 2); (2) Compared to the full AE model (Δ d.f. 1); (3) Compared to the AE model without sex-specific genetic effect (Δ d.f. 1).

**Supplementary Table 14.** Model fit statistics of body mass index (BMI) fluctuation (kg/m^2^ per year) in a pooled data with life-stage stratification

| **BMI fluctuation** | **Full ACE model (reference model)** | | **Full ADE model** | | **Full AE model (1)** | | **Selected model (AE) without sex-specific genetic effect (2)** | | **Selected model (AE) with same parameter estimates for men and women (3)** | |
| --- | --- | --- | --- | --- | --- | --- | --- | --- | --- | --- |
|  | **-2LL** | **d.f** | **-2LL** | **d.f** | **Δ -2 LL** | **p value** | **Δ -2 LL** | **p value** | **Δ -2 LL** | **p value** |
| *Young adulthood (18-30 years)* | 21621.09 | 2794 | 21619 | 2793 | 2.08 | 0.35 | 9.30 | 2.29e-03 | 60.93 | 5.84e-14 |
| *early midlife (31-50 years)* | 32279.44 | 3892 | 32289.21 | 3891 | 2.30 | 0.31 | 0.92 | 0.60 | 234.26 | 1.34e-51 |
| *Midlife (51-65 years)* | 19399.86 | 2272 | 19402.96 | 2271 | 0.01 | 1.00 | 0.64 | 0.42 | 104.68 | 1.85e-23 |
| *Older adulthood (≥66 years)* | 12075.23 | 1355 | 12075.32 | 1354 | 8.40e-03 | 0.99 | 0.16 | 0.96 | 5.75 | 0.04 |

**Caption:** Model comparisons were first conducted between the full ACE and ADE models based on the −2 log-likelihood (−2LL) values. The best-fitting model from this comparison was then tested against the simpler AE model. ACE model for all stages except young adulthood. Finally, the selected model was compared with (i) the same model without sex-specific genetic effects and (ii) the same model without sex differentiation. The table displays the −2 log-likelihood (−2LL) and degrees of freedom (df) for the reference model, as well as the differences in −2LL (Δ−2LL) and corresponding p-values for the compared models. **Abbreviations:** Δ (change); ACE (additive genetic/ shared environment/ unique environment) model; ADE (additive genetic/ dominance genetic/ unique environment); AE (additive genetic/ unique environment) model. (1) Compared to full ACE/ADE model (Δ d.f. 2); (2) Compared to the full AE model (Δ d.f. 1); (3) Compared to the AE model without sex-specific genetic effect (Δ d.f. 1).

**Supplementary Table 15.** Intraclass correlations (and 95% confidence interval) for body mass index (BMI) fluctuation (kg/m^2^ per year) in cohort specific data by sex and zygosity.

| **Cohorts** | **Men** | | | | | | **Women** | | | | | | **Opposite sex dizygotic (OSDZ)** | | |
| --- | --- | --- | --- | --- | --- | --- | --- | --- | --- | --- | --- | --- | --- | --- | --- |
|  | **MZ** | | | **DZ** | | | **MZ** | | | **DZ** | | |  |  |  |
|  | **r** | **95%CI** | | **r** | **95%CI** | | **r** | **95%CI** | | **r** | **95%CI** | | **r** | **95%CI** |  |
|  |  | **LL** | **UL** |  | **LL** | **UL** |  | **LL** | **UL** |  | **LL** | **UL** |  | **LL** | **UL** |
| *Danish Twin Cohort* | N= 443 | | | N= 386 | | | N= 555 | | | N= 519 | | | N= 638 | | |
|  | 0.44 | 0.36 | 0.51 | 0.08 | -0.01 | 0.18 | 0.12 | 0.04 | 0.20 | 0.08 | -0.02 | 0.15 | 0.03 | -0.04 | 0.11 |
| *Finnish old cohort* | N= 709 | | | N= 1,415 | | | N= 993 | | | N= 1,822 | | | N= 0 | | |
|  | 0.12 | 0.05 | 0.19 | 0.08 | 0.01 | 0.13 | 0.10 | 0.04 | 0.16 | 0.12 | 0.08 | 0.17 | - | - | - |
| *FinnTwin16* | N= 161 | | | N= 175 | | | N= 301 | | | N= 234 | | | N= 391 | | |
|  | 0.30 | 0.15 | 0.43 | 0.13 | -0.01 | 0.27 | 0.20 | 0.09 | 0.31 | 0.21 | 0.09 | 0.33 | -0.04 | -0.14 | 0.05 |
| *FinnTwin12* | N= 68 | | | N= 43 | | | N= 79 | | | N= 65 | | | N= 47 | | |
|  | 0.34 | 0.10 | 0.54 | 0.42 | 0.11 | 0.66 | 0.60 | 0.45 | 0.72 | 0.56 | 0.36 | 0.71 | 0.01 | -0.18 | 0.21 |
| *Italy Twin Registry* | N= 19 | | | N= 5 | | | N= 40 | | | N= 20 | | | N= 25 | | |
|  | 0.22 | -0.25 | 0.61 | 0.63 | -0.55 | 0.97 | 0.16 | -0.15 | 0.45 | 0.37 | -0.08 | 0.69 | -0.01 | -0.40 | 0.38 |
| *Netherlands Twin Register* | N= 339 | | | N= 175 | | | N= 849 | | | N= 379 | | | N= 407 | | |
|  | 0.155 | 0.05 | 0.25 | 0.13 | -0.01 | 0.27 | 0.20 | 0.14 | 0.27 | 0.02 | -0.07 | 0.12 | 0.15 | 0.05 | 0.24 |
| *Swedish Twin Registry* | N= 238 | | | N= 419 | | | N= 552 | | | N= 947 | | | N= 0 | | |
|  | 0.08 | -0.04 | 0.21 | 0.05 | -0.03 | 0.15 | 0.17 | 0.09 | 0.25 | 0.04 | -0.01 | 0.11 | - | - | - |
| *Swedish Young Male Twin Study* | N= 350 | | | N= 248 | | | N= 0 | | | N= 0 | | | N= 0 | | |
|  | 0.41 | 0.32 | 0.49 | 0.05 | -0.06 | 0.18 | - | - | - | - | - | - | - | - | - |
| *TwinsUK* | N= 64 | | | N= 50 | | | N= 777 | | | N= 728 | | | N= 6 | | |
|  | 0.12 | -0.12 | 0.35 | 0.25 | -0.03 | 0.49 | 0.22 | 0.16 | 0.29 | 0.13 | 0.06 | 0.20 | 0.39 | -0.61 | 0.91 |
| *Colorado Twin Registry* | N= 25 | | | N= 16 | | | N= 42 | | | N= 14 | | | N= 18 | | |
|  | -0.03 | -0.42 | 0.36 | 0.20 | -0.32 | 0.63 | 0.33 | 0.03 | 0.58 | -0.04 | -0.56 | 0.49 | -0.09 | -0.53 | 0.39 |
| *NAS-NRC Twin Registry* | N= 1,216 | | | N= 1,073 | | | N= 0 | | | N= 0 | | | N= 0 | | |
|  | 0.08 | 0.02 | 0.13 | 0.02 | -0.03 | 0.08 | - | - | - | - | - | - | - | - | - |
| *Washington State Twin Registry* | N= 245 | | | N= 84 | | | N= 574 | | | N= 202 | | | N= 179 | | |
|  | -0.01 | -0.13 | 0.12 | 0.29 | 0.08 | 0.48 | 0.30 | 0.22 | 0.37 | 0.19 | 0.05 | 0.32 | 0.12 | -0.02 | 0.26 |
| *Korea Twin-Family Register* | N= 37 | | | N= 12 | | | N= 115 | | | N= 18 | | | N= 0 | | |
|  | -0.16 | -0.46 | 0.17 | -0.16 | -0.67 | 0.45 | 0.01 | -0.17 | 0.18 | 0.40 | -0.07 | 0.73 | - | - | - |
| *Queensland Twin Register* | N= 308 | | | N= 242 | | | N= 662 | | | N= 457 | | | N= 498 | | |
|  | 0.10 | -0.01 | 0.21 | -0.02 | -0.15 | 0.10 | 0.18 | 0.10 | 0.25 | 0.17 | 0.08 | 0.26 | 0.06 | -0.02 | 0.15 |

**Abbreviations:** BMI, body mass index; CI, confidence interval; LL, lower limit; UL, upper limit; r: correlation coefficient; MZ: monozygotic; DZ: dizygotic; N: Number of complete twin pairs; UK: United Kingdom; NAS-NRC: National academy of science- National research council.

**Supplementary table 16.** Model fit statistics of body mass index (BMI) fluctuation (kg/m^2^ per year) in cohort specific data.

| **Cohorts** | **Full ACE model (reference model)** | | **Full ADE model** | | **Full AE model (1)** | | **Selected model (AE) without sex-specific genetic effect (2)** | | **Selected model (AE) with same parameter estimates for men and women (3)** | |
| --- | --- | --- | --- | --- | --- | --- | --- | --- | --- | --- |
|  | **-2LL** | **d.f** | **-2LL** | **d.f** | **Δ -2 LL** | **p value** | **Δ -2 LL** | **p value** | **Δ -2 LL** | **p value** |
| *Danish Twin Cohort* | 39247.15 | 5073 | 39244.23 | 5072 | 2.92 | 0.23 | 2.49 | 0.11 | 137.57 | 9.00e-32 |
| *Finnish old cohort* | 71205.96 | 9873 | 71211.64 | 9872 | 3.88 | 0.32 | 0.01 | 0.89 | 36.94 | 1.21e-09 |
| *FinnTwin16* | 18165.41 | 2515 | 18162.64 | 2514 | 4.02 | 0.13 | 20.28 | 6.66e-06 | 79.06 | 6.01e-19 |
| *FinnTwin12* | 7500.00 | 302 | 7512.60 | 301 | 0.13 | 0.93 | 13.5 | 7.84e-05 | 35.43 | 2.84e-11 |
| *Italy Twin Registry* | 1540.92 | 209 | 1541.80 | 208 | 0.87 | 0.64 | 0.28 | 0.62 | 45.20 | 1.77e-11 |
| *Netherlands Twin Register* | 37320.11 | 4296 | 37317.89 | 4295 | 2.39 | 0.30 | 1.84 | 0.17 | 5.03 | 0.02 |
| *Swedish Twin Registry* | 35684.70 | 4303 | 35682.13 | 4302 | 2.69 | 0.26 | 0.00 | 1.00 | 106.34 | 6.19e-25 |
| *Swedish Young Male Twin Study* | 8739.97 | 1192 | 8736.05 | 1191 | 7.82e-11 | 1.00 | - | - | - | - |
| *TwinsUK* | 29712.46 | 3241 | 29718.31 | 3240 | 1.96 | 0.37 | 1.02 | 0.31 | 5.8 | 0.04 |
| *Colorado Twin Registry* | 1564.45 | 221 | 1568.53 | 220 | 0.16 | 0.91 | 0.07 | 0.78 | 27.35 | 1.69e-07 |
| *NAS-NRC Twin Registry* | 38640.73 | 4573 | 38640.25 | 4572 | -9.76e-09 | 1.00 | - | - | - | - |
| *Washington State Twin Registry* | 21771.07 | 2559 | 21772.96 | 2558 | 1.88 | 0.39 | 0.36 | 0.54 | 20.41 | 6.24e-06 |
| *Korea Twin-Family Register* | 2575.90 | 355 | 2575.915 | 354 | 0.01 | 0.99 | 0.00 | 1.00 | 21.47 | 3.57e-06 |
| *Queensland Twin Register* | 37466.72 | 4325 | 37468.54 | 4324 | 2.38 | 0.30 | 0.02 | 0.87 | 5.93 | 0.01 |

**Caption:** Model comparisons were first conducted between the full ACE and ADE models based on the −2 log-likelihood (−2LL) values. The best-fitting model from this comparison was then tested against the simpler AE model. ACE was selected for Finnish old cohort, FinnTwin12, Italy Twin Registry, TwinsUK, Colorado Twin Registry, Washington State Twin Registry, Korea Twin-Family Register and Queensland Twin Register. Finally, the selected model (AE for all the cohorts) was compared with (i) the same model without sex-specific genetic effects and (ii) the same model without sex differentiation. The table displays the −2 log-likelihood (−2LL) and degrees of freedom (df) for the reference model, as well as the differences in −2LL (Δ−2LL) and corresponding p-values for the compared models. **Abbreviations:** Δ (change); ACE (additive genetic/ shared environment/ unique environment) model; ADE (additive genetic/ dominance genetic/ unique environment); AE (additive genetic/ unique environment) model. (1) Compared to full ACE/ADE model (Δ d.f. 2); (2) Compared to the full AE model (Δ d.f. 1); (3) Compared to the AE model without sex-specific genetic effect (Δ d.f. 1); UK: United Kingdom; NAS-NRC: National academy of science- National research council.

**Supplementary Table 17.** Heritability and relative proportion of unique environmental variance for body mass index (BMI) fluctuation rate (kg/m² per year) with 95% confidence intervals, presented for each cohort and stratified by sex.

| **Cohorts** | **Men** | | | | | | **Women** | | | | | |
| --- | --- | --- | --- | --- | --- | --- | --- | --- | --- | --- | --- | --- |
|  | **a^2^** | **95% CI** | | **e^2^** | **95% CI** | | **a^2^** | **95% CI** | | **e^2^** | **95% CI** | |
|  |  | **LL** | **UL** |  | **LL** | **UL** |  | **LL** | **UL** |  | **LL** | **UL** |
| *Danish Twin Cohort* | 0.38 | 0.31 | 0.45 | 0.62 | 0.54 | 0.68 | 0.11 | 0.03 | 0.17 | 0.89 | 0.82 | 0.96 |
| *Finnish old cohort* | 0.14 | 0.07 | 0.20 | 0.86 | 0.79 | 0.92 | 0.15 | 0.10 | 0.19 | 0.85 | 0.80 | 0.89 |
| *FinnTwin16* | 0.57 | 0.41 | 0.67 | 0.43 | 0.32 | 0.58 | 0.26 | 0.15 | 0.35 | 0.74 | 0.65 | 0.84 |
| *FinnTwin12* | 0.37 | 0.14 | 0.46 | 0.63 | 0.46 | 0.75 | 0.29 | 0.18 | 0.40 | 0.71 | 0.60 | 0.81 |
| *Italy Twin Registry* | 0.24 | 0.01 | 0.58 | 0.76 | 0.41 | 1.00 | 0.16 | 0.01 | 0.38 | 0.84 | 0.61 | 1.00 |
| *Netherlands Twin Register* | 0.20 | 0.09 | 0.28 | 0.80 | 0.71 | 0.90 | 0.20 | 0.13 | 0.25 | 0.80 | 0.74 | 0.86 |
| *Swedish Twin Registry* | 0.09 | 0.01 | 0.18 | 0.91 | 0.81 | 1.00 | 0.16 | 0.08 | 0.23 | 0.84 | 0.76 | 0.91 |
| *Swedish Young Male Twin Study* | 0.37 | 0.28 | 0.45 | 0.63 | 0.54 | 0.71 | - | - | - | - | - | - |
| *Twins UK* | 0.15 | 0.01 | 0.34 | 0.85 | 0.66 | 0.99 | 0.25 | 0.18 | 0.30 | 0.75 | 0.69 | 0.81 |
| *Colorado Twin Registry* | 0.10 | 0.01 | 0.45 | 0.90 | 0.54 | 1.00 | 0.29 | 0.01 | 0.51 | 0.71 | 0.48 | 0.98 |
| *NAS-NRC Twin Registry* | 0.08 | 0.02 | 0.13 | 0.92 | 0.86 | 0.97 | - | - | - | - | - | - |
| *Washington State Twin Registry* | 0.06 | 0.01 | 0.17 | 0.94 | 0.82 | 1.00 | 0.29 | 0.21 | 0.35 | 0.71 | 0.64 | 0.78 |
| *Korea Twin-Family Register* | 0.07 | 0.01 | 0.18 | 0.93 | 0.82 | 1.00 | 0.26 | 0.06 | 0.46 | 0.74 | 0.45 | 1.00 |
| *Queensland Twin Register* | 0.08 | 0.01 | 0.17 | 0.92 | 0.82 | 0.99 | 0.21 | 0.14 | 0.27 | 0.79 | 0.72 | 0.85 |

**Caption:** AE model without sex specific genetic effect was selected for BMI fluctuation in all the cohorts except FinnTwin12, FinnTwin16, Swedish Young Male Twin Study and NAS-NRC Twin Registry where full AE model was selected. **Abbreviations:** a^2^: Heritability (proportion of total variance explained by additive genetic factors); e^2^: proportion of total variance explained by unique environmental factors; CI: Confidence interval; LL: Lower limit; UL: Upper limit; UK: United Kingdom; NAS-NRC: National Academy of Science- National Research Council.

| **Life stages** | **Men** | | | | | | **Women** | | | | | | |
| --- | --- | --- | --- | --- | --- | --- | --- | --- | --- | --- | --- | --- | --- |
|  | **r_A_** | **95% CI** | | **r_E_** | **95% CI** | | **r_A_** | **95% CI** | | **r_E_** | **95% CI** | |  |
|  |  | **LL** | **UL** |  | **LL** | **UL** |  | **UL** | **LL** |  | **LL** | **UL** |  |
| *Young adulthood*  *(18-30 years)* | | | | | | | | | | | | | |
| Baseline BMI | 0.49 | 0.41 | 0.57 | 0.25 | 0.17 | 0.33 | 0.52 | 0.39 | 0.65 | 0.25 | 0.14 | 0.35 |  |
| BMI change | 0.02 | -0.10 | 0.15 | -0.12 | -0.20 | -0.03 | 0.05 | -0.12 | 0.26 | -0.07 | -0.17 | 0.03 |  |
| *Early midlife*  *(31-50 years)* | | | | | | | | | | | | | |
| Baseline BMI | 0.49 | 0.37 | 0.61 | 0.17 | 0.06 | 0.28 | 0.31 | 0.27 | 0.34 | 0.50 | 0.39 | 0.62 |  |
| BMI change | 0.13 | -0.01 | 0.28 | -0.07 | -0.19 | 0.04 | 0.06 | 0.02 | 0.10 | 0.31 | 0.17 | 0.45 |  |
| *Midlife*  *(51-65 years)* | | | | | | | | | | | | | |
| Baseline BMI | -0.03 | -0.38 | 0.28 | 0.35 | 0.22 | 0.49 | 0.18 | -0.02 | 0.37 | 0.20 | 0.03 | 0.38 |  |
| BMI change | -0.19 | -0.58 | 0.22 | 0.09 | -0.05 | 0.23 | 0.08 | -0.17 | 0.39 | -0.11 | -0.34 | 0.15 |  |
| *Older adulthood*  *(>66 years)* | | | | | | | | | | | | | |
| Baseline BMI | 0.10 | -0.15 | 0.35 | 0.27 | 0.14 | 0.40 | -0.03 | -0.34 | 0.28 | 0.35 | 0.24 | 0.46 |  |
| BMI change | -0.14 | -0.42 | 0.15 | 4.00e-03 | -0.14 | 0.14 | -0.02 | -0.20 | 0.16 | -5.00e-03 | -0.14 | 0.13 |  |

**Supplementary Table 18.** Additive genetic and unique environmental correlations of the associations between BMI fluctuation and BMI trajectories with life-stage stratification in the pooled data by sex.

**Caption**: Pairwise correlations between BMI fluctuation and BMI trajectories with life-stage stratification were carried out. They are summarized with correlation estimates besides their confidence intervals. Abbreviations: r_A_: additive genetic correlation; r_E_; specific environmental correlation; CI: Confidence interval; LL: lower limit; UL: upper limit; BMI: body mass index

**Methodological appendix**

**Supplementary Table 19.** Comparison of intraclass correlations for body mass index (BMI) fluctuation (kg/m^2^ per year) between the full pooled sample and the life-stage–restricted sample.

| **BMI fluctuation** | **Men** | | | | | | **Women** | | | | | | | **Opposite sex dizygotic (OSDZ)** | | |
| --- | --- | --- | --- | --- | --- | --- | --- | --- | --- | --- | --- | --- | --- | --- | --- | --- |
|  | **MZ** | | | **DZ** | | | **MZ** | | | **DZ** | | | |  |  |  |
|  | **r** | **95%CI** | | **r** | **95%CI** | | **r** | **95%CI** | | **r** | **95%CI** | | **r** | | **95%CI** | |
|  |  | **LL** | **UL** |  | **LL** | **UL** |  | **LL** | **UL** |  | **LL** | **UL** |  |  | **LL** | **UL** |
| *Full pooled sample* | N= 4,156 | | | N= 4,301 | | | N= 5,519 | | | N= 5,394 | | | | N= 2,164 | | |
|  | 0.18 | 0.15 | 0.21 | 0.11 | 0.08 | 0.14 | 0.26 | 0.24 | 0.29 | 0.21 | 0.19 | 0.24 | 0.13 | | 0.09 | 0.17 |
| *Life-stage restricted sample* | N= 1,029 | | | N= 803 | | | N= 1,627 | | | N= 1,270 | | | N= 449 | | | |
|  | 0.34 | 0.29 | 0.39 | 0.11 | 0.04 | 0.17 | 0.22 | 0.17 | 0.26 | 0.22 | 0.17 | 0.27 | 0.02 | | -0.07 | 0.11 |

**Caption**: Intraclass correlations for BMI fluctuation full pooled sample and the life-stage–restricted sample are summarized by sex and zygosity with correlation coefficients besides their 95% confidence interval. **Abbreviations:** BMI, body mass index; CI, confidence interval; LL, lower limit; UL, upper limit; r: correlation coefficient; MZ: monozygotic; DZ: same sex dizygotic; OSDZ: opposite sex dizygotic; N: Number of complete twin pairs.

**Supplementary Table 20.** Comparison of model fit statistics of body mass index (BMI) fluctuation (kg/m^2^ per year) between the full pooled sample and the life-stage–restricted sample.

| **BMI fluctuation** | **Full ACE model (reference model)** | | **Full ADE model** | | **Full AE model (1)** | | **Selected model without sex-specific genetic effect (2)** | | **Selected model with same parameter estimates for men and women (3)** | |
| --- | --- | --- | --- | --- | --- | --- | --- | --- | --- | --- |
|  | **-2LL** | **d.f** | **-2LL** | **d.f** | **Δ -2 LL** | **p value** | **Δ -2 LL** | **p value** | **Δ -2 LL** | **p value** |
| *Full pooled sample* | 357546.3 | 43049 | 357580.2 | 43048 | 3.38 | 0.44 | 0.49 | 0.48 | 71.53 | 1.36e-15 |
| *Life-stage restricted sample* | 78894.07 | 9442 | 78905.79 | 9441 | 12.13 | 0.23 | 0 | 1 | 495.21 | 1.04e-109 |

**Caption:** Model fit statistics for BMI fluctuation are presented separately for the full pooled sample and the life-stage–restricted sample. For each sample, model comparisons were first conducted between the full ACE and ADE models based on −2 log-likelihood (−2LL) values. The better-fitting model from this comparison was subsequently tested against the more parsimonious AE model. Finally, the selected model was compared with (i) the same model without sex-specific genetic effects and (ii) the same model with equal parameter estimates for men and women. The table reports −2LL values, degrees of freedom (df), differences in −2LL (Δ−2LL), and corresponding p-values for nested model comparisons. **Abbreviations:** Δ, change; ACE, additive genetic/shared environmental/unique environmental model; ADE, additive genetic/dominance genetic/unique environmental model; AE, additive genetic/unique environmental model; −2LL, −2 log-likelihood; df, degrees of freedom. (1) Compared with the full ACE model (Δdf = 2); (2) compared with the full AE model (Δdf = 1); (3) compared with the AE model without sex-specific genetic effects (Δdf = 1).

**Supplementary Table 21.** Comparison of Heritability and relative proportion of unique environmental variance for body mass index (BMI) fluctuation rate (kg/m² per year) between full pooled sample and the life-stage–restricted sample in both cases stratified by sex.

**Caption:** Full AE model was selected for BMI fluctuation in both full pooled sample and the life-stage–restricted sample. **Abbreviations:** a^2^: Heritability (proportion of total variance explained by additive genetic effects); e^2^: proportion of total variance explained by unique environmental effects; CI: Confidence interval; LL: Lower limit; UL: Upper limit.

| **BMI fluctuation** | **Men** | | | | | | **Women** | | | | | |
| --- | --- | --- | --- | --- | --- | --- | --- | --- | --- | --- | --- | --- |
|  | **a^2^** | **95% CI** | | **e^2^** | **95% CI** | | **a^2^** | **95% CI** | | **e^2^** | **95% CI** | |
|  |  | **LL** | **UL** |  | **LL** | **UL** |  | **LL** | **UL** |  | **LL** | **UL** |
| Pooled data without life-stage stratification | | | | | | | | | | | | |
| *Full pooled sample* | 0.20 | 0.17 | 0.22 | 0.80 | 0.77 | 0.82 | 0.29 | 0.26 | 0.31 | 0.71 | 0.68 | 0.73 |
| *Life-stage restricted sample* | 0.31 | 0.26 | 0.36 | 0.69 | 0.64 | 0.73 | 0.27 | 0.22 | 0.31 | 0.73 | 0.69 | 0.77 |

**Comparison of Twin Correlations and Model Selection Between the Full Sample and the Life-Stage–Restricted Subset**

To assess the robustness of our results across age-restricted analyses, we compared intraclass correlations (ICCs), model fit statistics, and heritability estimates for BMI fluctuation between the full pooled sample and the life-stage–restricted subset. The subset included only twin pairs whose first and last BMI measurements fell within the same life stage (young adulthood, middle–late adulthood, middle age, or older age), representing less than one quarter of all pairs.

ICCs are shown in Supplementary Table 19. In men, MZ twin correlations increased markedly in the life-stage–restricted subset (r = 0.18 in the full pooled sample vs 0.34 in the subset), whereas DZ correlations remained stable (r = 0.11 vs 0.11). In women, both MZ and DZ correlations were largely unchanged (MZ: 0.26 vs 0.22; DZ: 0.21 vs 0.22), For opposite-sex dizygotic (OSDZ) pairs, correlations decreased substantially from the full pooled sample to the life-stage–restricted subset (r = 0.13 vs 0.02), with the correlation in the subset no longer statistically significant. This pattern reflects reduced similarity in BMI fluctuation between opposite-sex pairs in the age-restricted analysis. The increase in MZ correlations among men indicates that restricting the sample by life stage reduced within-pair variability in ways that disproportionately affected genetically identical twins.

Model fit comparisons are presented in Supplementary Table 20. In men, the observed change in twin correlations led to a shift from the ACE model (additive genetic, shared environment, unique environment) to the ADE model (additive + dominance genetic, unique environment), reflecting a stronger contribution of dominance genetic effects in the restricted sample. In women, model selection remained unchanged, consistent with the minimal change in correlations. For both sexes, the AE model continued to provide a reasonable fit when sex-specific effects were not included.

Heritability and unique environmental variance estimates are summarized in Supplementary Table 21. In men, additive genetic effects (a²) increased from 0.20 in the full pooled sample to 0.31 in the life-stage–restricted subset, while in women heritability remained similar (0.29 vs 0.27). Unique environmental effects (e²) remained the predominant source of variance across both sexes and samples (men: from 0.80 to 0.69; women: from 0.71 to 0.73), highlighting the major role of individual-specific environmental factors in BMI fluctuation.

Overall, these results suggest that restricting the sample by life stage slightly amplified the genetic contribution to BMI fluctuation in men, primarily due to increased MZ correlations, while leaving female estimates largely unchanged. The predominance of unique environmental variance across both sexes and samples indicates that individual-specific experiences and lifestyle factors remain the major determinants of BMI fluctuation. These sensitivity analyses confirm that the main findings in the full pooled sample are robust, and that observed differences in the restricted subset likely reflect changes in variance structure and sample composition rather than substantive alterations in the underlying genetic architecture.
